# instaGRAAL: chromosome-level quality scaffolding of genomes using a proximity ligation-based scaffolder

**DOI:** 10.1186/s13059-020-02041-z

**Published:** 2020-06-18

**Authors:** Lyam Baudry, Nadège Guiglielmoni, Hervé Marie-Nelly, Alexandre Cormier, Martial Marbouty, Komlan Avia, Yann Loe Mie, Olivier Godfroy, Lieven Sterck, J. Mark Cock, Christophe Zimmer, Susana M. Coelho, Romain Koszul

**Affiliations:** 1 Institut Pasteur, Unité Régulation Spatiale des Génomes, CNRS, UMR 3525, C3BI USR 3756, F-75015 Paris, France; 2 Sorbonne Université, Collège Doctoral, F-75005 Paris, France; 3 Evolutionary Biology & Ecology, Université Libre de Bruxelles, 1050 Brussels, Belgium; 4 Sorbonne Université, Laboratory of Integrative Biology of Marine Models, Algal Genetics, UMR 8227, Roscoff, France; 5 Present Address: Université de Strasbourg, INRA, SVQV UMR-A 1131, Colmar, France; 6 Institut Pasteur, Center of Bioinformatics, Biostatistics and Integrative Biology (C3BI), USR3756, CNRS, Paris, France; 7 Department of Plant Biotechnology and Bioinformatics, Ghent University, B-9052 Ghent Ghent, Belgium; 8 VIB Center for Plant Systems Biology, Technologiepark 927, B-9052 Ghent, Belgium; 9 Institut Pasteur, Imaging and Modeling Unit, CNRS, UMR 3691, C3BI USR 3756, F-75015 Paris, France

**Keywords:** *Ectocarpus*, Hi-C scaffolding, Hi-C, genome assembly, MCMC, GPU, *Desmarestia herbacea*

## Abstract

Hi-C exploits contact frequencies between pairs of loci to bridge and order contigs during genome assembly, resulting in chromosome-level assemblies. Because few robust programs are available for this type of data, we developed instaGRAAL, a complete overhaul of the GRAAL program, which has adapted the latter to allow efficient assembly of large genomes. instaGRAAL features a number of improvements over GRAAL, including a modular correction approach that optionally integrates independent data. We validate the program using data for two brown algae, and human, to generate near-complete assemblies with minimal human intervention.

## Background

Continuous developments in DNA sequencing technologies aim at alleviating the technical challenges that limit the ability to assemble sequence data into full-length chromosomes [1–3]. Conventional assembly programs and pipelines often encounter difficulties to close gaps in draft genome assemblies introduced by regions enriched in repeated elements. These assemblers efficiently generate overlapping sets of reads (i.e., contiguous sequences or contigs) but encounter difficulties linking these contigs together into scaffolds. At the chromosome level, these programs often incorrectly orient DNA sequences or predict incorrect numbers of chromosomes [4]. The development of long-read sequencing technology and accompanying assembly programs has considerably alleviated these difficulties, but some gaps remain nevertheless in genome scaffolds, notably at the level of long repeated/low-complexity DNA sequences. In addition, long-read-based assemblies are associated with increased error rate among long reads, which can result in misassemblies [3]. Consequently, many currently available genomes still contain structural errors, as well as gaps that need to be bridged to reach a chromosome-level structure.

These limitations have been partially addressed thanks to active support from the community and competitions such as GAGE [5] or the Assemblathon [6]. However, there is as yet no systematic, reliable workflow of producing near-perfect genome assemblies of guaranteed optimal best quality without a considerable amount of empiric parameter adjustment and manual post-processing evaluation and correction [7].

Recent sequencing projects have typically relied on a combination of independently obtained data such as optical mapping, long-read sequencing, and chromosomal conformation capture (3C, Hi-C) to obtain large genome assemblies of high accuracy. The latter procedure derives from techniques aiming at recovering snapshots of the higher-order organization of a genome [8, 9]. When applied to genomics, Hi-C-based methods are sometimes referred to as proximity ligation approaches, as they quantify and exploit physical contacts between pairs of DNA segments in a genome to assess their collinearity along a chromosome, and the distance between the segments [10]. Early studies using control datasets demonstrated that Hi-C can be used to scaffold and/or correct a wide range of eukaryotic DNA regions [11–14], i.e. stretches of bp, whether they be small-scale contigs or full chromosomes. The Hi-C scaffolder GRAAL (Genome Re-Assembly Assessing Likelihood from 3D) is a probabilistic program that uses a Markov Chain Monte Carlo (MCMC) approach. This tool was able to generate the first chromosome-level assembly of an incomplete eukaryote genome [13] by permuting DNA segments according to their contact frequencies until the most likely scaffold was reached (see also [15]). Since these proof of concept studies, the assemblies of many genomes of various sizes from eukaryotes [16–18] and prokaryotes [19] have been significantly improved using scaffolding approaches exploiting Hi-C data.

Although GRAAL was effective on medium-sized or small (< 100 Mb) eukaryotic genomes such as that of the fungus *Trichoderma reesei* [20], scalability limitations were encountered when tackling genomes whose complexity and size required significant computer calculation capacity. Furthermore, as was also observed with other Hi-C-based scaffolders, the raw output of GRAAL includes a number of caveats that need to be corrected manually to obtain a finished genome assembly. To overcome these limitations, we developed instaGRAAL, an enhanced, open-source program optimized to reduce the computational load of chromosome scaffolding and that includes a misassembly “correction” module installed alongside the scaffolder. Moreover, instaGRAAL can optionally exploit available genetic linkage data.

We applied instaGRAAL to three genomes of increasing size: in the first two runs, and in order to demonstrate its added value, we applied the program to the 214-Mb and 500-Mb haploid genomes of the brown alga *Ectocarpus* sp. [21, 22] and *Desmarestia herbacea* (unpublished), respectively. Brown algae are a group of complex multicellular eukaryotes that have been evolving independently from animal and land plants for more than a billion years. *Ectocarpus* sp. was the first species within the brown algal group to be sequenced (reference v1 assembly [22]), as a model organism to investigate multiple aspects of brown algal biology including the acquisition of multicellularity, sex determination, life cycle regulation, and adaptation to the intertidal [22–25]. A range of genetic and genomic resources have also been established for *Ectocarpus* sp. including a dense genetic map generated with 3588 SNP markers (v2 assembly) [26], which was used to comprehensively validate both a GRAAL (v3) and the instaGRAAL (v4) assemblies. In a third run, we benchmarked instaGRAAL using the human genome, to confirm that our software readily scales to larger (Gb-sized) and more complex assemblies, an important requirement to tackle the next era of assembly projects.

## Results

### From GRAAL to instaGRAAL

The core principles of GRAAL and instaGRAAL are similar: both exploit a MCMC approach to perform a series of permutations (insertions, deletions, inversions, swapping, etc.) of genome fragments (referred to here as “bins,” see the “Material and methods” section) based on an expected contact distribution [13]. The parameters (*A*, *α*, and *δ*) that describe this contact distribution are first initialized using a model inspired by polymer physics [27]. This model describes the expected contact frequency *P*(*s*) between two loci separated by a genomic distance *s* (when applicable):

$$ P(s)=\left\{\begin{array}{c}\max \left(A\cdotp {s}^{-\alpha },\delta \right):\in \mathrm{tracontacts}\\ {}\delta :\mathrm{intercontacts}\end{array}\right. $$

The parameters are then iteratively updated directly from the real scaffolds once their sizes increase sufficiently [13]. Each bin is tested in several positions relative to putative neighboring fragments. The likelihood of each arrangement is assessed from the simulated or computed contact distribution, and the arrangement is either accepted or rejected [13]. This analysis is carried out in cycles, with a cycle being completed when all the bins of the genome have been processed in this way. Any number of cycles can be run iteratively, and the process is usually continued until the genome structure ceases to evolve, as measured by the evolution of the parameters of the model. The core functions of the program use Python libraries, as well as the CUDA programming language, and therefore necessitate a NVIDIA graphics card with at least 1 Gb of memory.

The technical limitations of GRAAL were (1) high memory usage when handling Hi-C data for large genomes (i.e. over 100 Mb), (2) difficulties when installing the software, and (3) the need to adjust multiple ad hoc parameters to adapt to differences in genome size, read coverage, Hi-C contact distribution, specific contact features, etc. instaGRAAL (https://github.com/koszullab/instaGRAAL) addresses all these shortcomings. First, we rewrote the memory-critical parts of the program, such as permutation sampling and likelihood calculation, so that they are computed using sparse contact maps. We reduced the software’s dependency footprint and added detailed documentation, deployment scripts, and containers to ease its installation. Finally, we opened up multiple hard-coded parameters to give more control for end-users while improving the documentation on each of them and selecting relevant default parameters that can be implemented for a wide range of applications (see options online and the “Discussion” section). Overall, these upgrades result in a program that is lighter in resources, more flexible, and more user-friendly.

Other problems encountered with the original GRAAL program included (1) the presence of potential artifacts introduced by the permutation sampler, such as spurious permutations (e.g. local inversions) or incorrect junctions between bins; (2) difficulties with the correct integration of other types of data such as long reads; and (3) difficulties with handling sequences that were either too short, highly repeated, or with low coverage. We addressed these points by identifying and putting aside these problematic sequences during a filtering step. These sequences are subsequently reinserted into the final scaffolds, whenever possible (see the “Material and methods” section), with the help of linkage data when available. Overall, when compared to the raw GRAAL output, the resulting “corrected” instaGRAAL assemblies were significantly more complete and more faithful to the actual chromosome structure.

### Scaffolding of the *Ectocarpus* sp. chromosomes with instaGRAAL

**Fig. 1:**
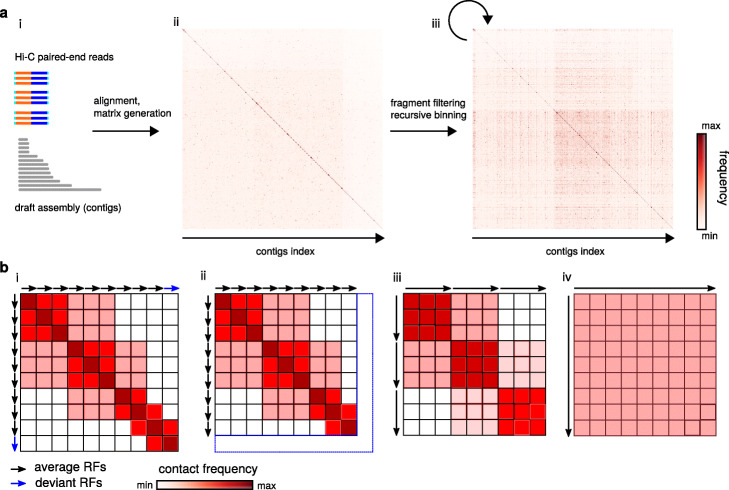
Matrix generation and binning process. **a** From left to right: (i) the input data to be processed, and paired-end reads to be mapped onto the *Ectocarpus*. sp. reference v1 genome assembly; (ii) raw contact map before binning—each pixel is a contact count between two restriction fragments (RF); and (iii) raw contact map after binning—each pixel is a contact between a determined number of RFs (see **b**). **b** Schematic description of one iteration of the binning process over 10 restriction fragments (arrows). From left to right: (i) initial contact map, each pixel is a contact count between two RFs; (ii) filtering step—RFs either too short or presenting a read coverage below one standard deviation below the mean are discarded; (iii) binning step (1 bin = 3RFs)—adjacent RFs are pooled by three, with sum-pooling along all pixels in a 3 × 3 square; and (iv) binning step (1 bin = 9 RFs)—adjacent RFs are pooled by nine

To test and validate instaGRAAL, we generated an improved assembly of the genome of the model brown alga *Ectocarpus* sp. A v1 genome consisting of 1561 scaffolds generated from Sanger sequence data is available [22]. A Hi-C library was generated from a clonal culture of a haploid partheno-sporophyte carrying the male sex chromosome using a GC-neutral restriction enzyme (DpnII). The library was paired-end sequenced (2 × 75 bp—the first ten bases were used as a tag and to remove PCR duplicates) on a NextSeq apparatus (Illumina). Of the resulting 80,521,968 paired-end reads, 41,288,678 read pairs were aligned unambiguously along the v1 genome using bowtie2 (quality scores below 30 were discarded), resulting in 2,554,639 links bridging 1,806,386 restriction fragments (Fig. 1a) (see the “Material and methods” section for details on the experimental and computational steps). The resulting contact map in sparse matrix format was then used to initialize instaGRAAL along with the restriction fragments (RFs) of the reference genome (Fig. 1a, b) (see Additional file 1: Table S1 for an example of sparse file matrix).

**Fig. 2:**
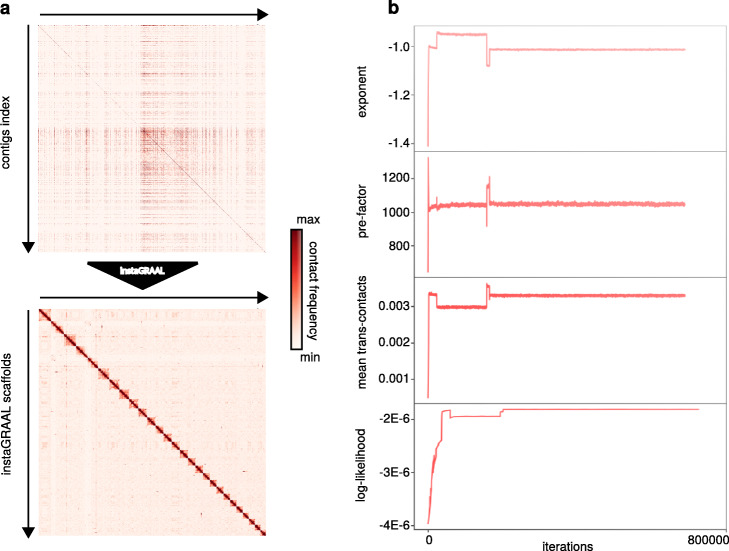
Evolution of the *Ectocarpus* sp. contact map, the parameters of the polymer model, and the log-likelihood of the contact map. **a** The raw contact map before (upper part) and after (bottom part) scaffolding using instaGRAAL. Scaffolds are ordered by size. **b** Evolution of three parameters of the polymer model (exponent, pre-factor, mean trans-contacts) and the log-likelihood as a function of iterations

Given the probabilistic nature of the algorithm, we evaluated the program’s consistency by running it three times with different resolutions. Briefly, we filtered out RFs that were shorter than 50 bp and/or whose coverage was one standard deviation below the mean coverage. Then, we sum-pooled (or binned) the sparse matrix by groups (or bins) of three RFs five times, recursively (Fig. 1a, b). Each recursive instance of the sum-pooling is subsequently referred to as a level of the contact map. A level determines the resolution at which permutations are being tested: the higher the level, the lower the resolution, the longer the sequences being permuted and, consequently, the faster the computation. The binning process is shown in Fig. 1b. Regarding *Ectocarpus* sp., we found that level 4 (bins of 81 RFs) was an acceptable balance between high resolution and fast computation on a desktop computer with a GeForce GTX TITAN Z graphics card. Moreover, whether instaGRAAL was run at level 4, 5, or 6 (equivalent to bins of 81, 243, and 729 RFs, respectively), all assemblies quickly (~ 6 h) converged towards similar genome structures (Fig. 2a).

**Fig. 3:**
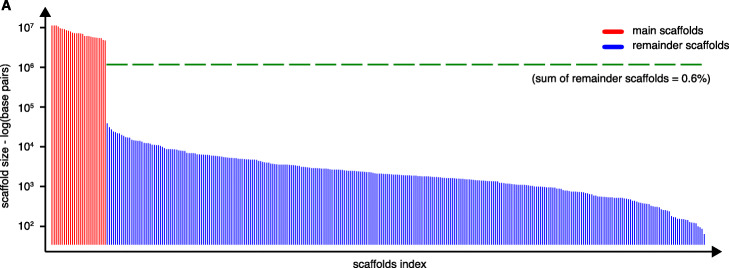
Size distribution (log scale) of the final *Ectocarpus* sp. scaffolds after 250 instaGRAAL iterations. After filtering, and prior to correction, 27 main scaffolds (red bars) or putative chromosomes were obtained. The dotted green horizontal line represents the proportion of the filtered genome that was not integrated into the main 27 scaffolds and represents less than 0.6% of the initial assembly. Each scaffold presents, after normalization, a high-quality Hi-C profile with features that are typical of eukaryotic genomes (Additional file 1 Fig. S1)

We plotted the evolution of the log-likelihood and of model parameters as a function of the number of arrangements performed (iterations) (Fig. 2b). The interquartile ranges (IQR, used to indicate stability in Marie-Nelly et al. [13]) of all parameters decreased to near-zero values at the end of each scaffolding run, indicating that they all stably converged and that the final structures oscillated near the final values in negligible ways. More qualitatively, each run led to the formation of 27 main scaffolds (Fig. 2a) with the 27th largest scaffold being more than a hundred times longer than the 28th largest one (Fig. 3, Additional file 1: movie S1). Each of the 27 scaffolds was between four and ten times longer than the combined length of the remaining sequences (Fig. 3). This strongly suggests that the 27 scaffolds correspond to chromosomes, a number consistent with karyotype analyses [28]. Taken together, these results indicate that instaGRAAL successfully assembled the *Ectocarpus* sp. genome into chromosome-level scaffolds. As the supplementary movie suggests, scaffold-level convergence is visible after only a few cycles, indicating that instaGRAAL is able to quickly determine the global genome structure most likely to fit the contact data. The remainder of the cycles is devoted to intra-chromosomal refinement.

### Correcting the chromosome-level instaGRAAL assembly of the *Ectocarpus* sp. genome

**Fig. 4:**
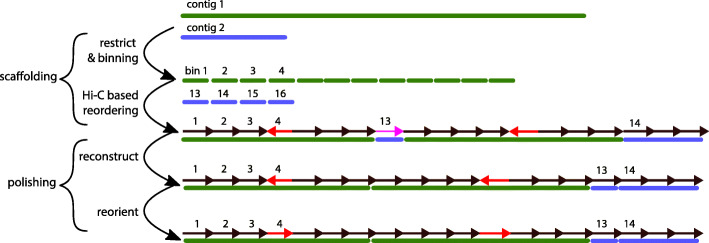
Step-by-step correction procedure. Correction procedure (top to bottom): (i) in silico restriction of the genome and binning, yielding a set of bins; (ii) reordering of all bins into scaffolds without taking into account their input contig of origin; typically, groups of bins from the same input contig naturally aggregate, but some bins get scattered to other scaffolds (e.g., bin 13, pink arrow), while others will be “flipped” with respect to the original assembly (e.g., bin 4, red arrows); (iii) reconstruction of the original input contigs by relocating scattered bins next to the biggest bin group; and (iv) bins in the original input contigs are oriented according to their original consensus orientation

instaGRAAL also includes a number of procedures that aim to correct some of the modifications introduced into the input contigs from the original assembly by the Hi-C scaffolding (Fig. 4). We implemented it as a separate “correction” module that is automatically installed alongside the scaffolder.

These modifications principally involve discrete inversions or insertions of DNA segments (typically corresponding to single bins or RFs) (see also [13]). Such alterations are inherent to the statistical nature of instaGRAAL, which will occasionally improperly permute neighboring bins because of the high density of contacts between them. However, we reasoned that input contigs from the original assembly, especially those generated for *Ectocarpus* sp. with Sanger sequencing, were unlikely to contain misassemblies. Therefore, we decided to favor input contigs’ structure whenever local conflicts arose. These are part of a broader set of assembly errors that we detected by aligning the v1 assembly on the instaGRAAL scaffolds and analyzing the mapping results using QUAST. The v1 assembly was used as a reference by QUAST to identify potential errors introduced by instaGRAAL when scaffolding the v1 assembly. We corrected these errors as follows: first, all bins processed by instaGRAAL that belonged to the same input contig were constrained to their original orientation (Fig. 4). If an input contig was split across multiple scaffolds, the smaller parts of this contig were relocated to the largest one, respecting the original order and orientation of the bins. Then, we reinserted whenever possible sequences that had been filtered out prior to instaGRAAL processing (e.g., contig extremities with poor read coverage; see the “Material and methods” section and Marie-Nelly et al. [13]) into the chromosome-level scaffold at their original position in the original input contig. 3,832,980 bp were reinserted into the assembly this way. These simple steps alleviated artificial truncations of input contigs observed with the original GRAAL program.

Some filtered bins had no reliable region to be associated with post-scaffolding, because their initial input contig had been completely filtered before scaffolding. These sequences, which were left as-is and appended at the end of the genome, were included into 543 scaffolds spanning 3,141,370 bp, i.e., < 2% of the total DNA. Together, these steps removed all the misassemblies detected by QUAST.

To further validate the assembly, we exploited an assembly generated by combining genetic recombination data and the Sanger assembly [21, 26] (“linkage group [LG] v2 assembly”) as well as an assembly generated by running the original GRAAL program on the original reference v1 genome assembly (“GRAAL v3 assembly”).

We searched for potential translocations between scaffold extremities between the linkage group v2 assembly and the v3 or v4 assemblies. This comparison, which was implemented as a separate module installed alongside the scaffolder, detected such events in the uncorrected v3 GRAAL assembly but none in the corrected v4 instaGRAAL assembly. The corrected instaGRAAL v4 assembly is therefore fully consistent with the genetic recombination map data, confirming the efficiency of the approach.

### Comparisons with previous *Ectocarpus* sp. assemblies and validation of the instaGRAAL assembly

We compared the corrected instaGRAAL v4 assembly with the three earlier assemblies of the *Ectocarpus* sp. genome mentioned above (Table 1 and Additional file 1: Table S2): (1) the original v1 genome assembly generated using Sanger sequencing data [22], which was assumed to be highly accurate but fragmented (1561 scaffolds); (2) the linkage group [LG] v2 assembly; and (3) the original GRAAL program v3 assembly.

**Table 1:** Comparison of Nx, NGx (i.e., Nx with respect to the original reference v1 genome assembly; in bp), and BUSCO completeness for the different assemblies (linkage group v2, GRAAL v3, and corrected instaGRAAL v4) of the *Ectocarpus* sp. genome

	Reference v1 assembly	Linkage group v2 assembly	v3 GRAAL	v4 corrected instaGRAAL
N50	497,380	6,528,661	6,867,074	6,813,345
NG50	497,380	6,528,661	6,725,743	6,813,345
N75	233,412	5,613,161	5,693,784	5,686,617
NG75	233,412	5,613,161	5,672,622	5,686,617
L50	118	12	11	11
LG50	118	12	12	11
L75	258	19	18	19
LG75	258	19	19	19
BUSCO completeness (%)	75.9	76.9	76.24	77.56

We aligned the corrected instaGRAAL (v4), LG (v2), and GRAAL (v3) assemblies onto the original v1 assembly to detect misassemblies and determine whether the genome annotations (362,919 features) were conserved. We then validated each assembly using genetic linkage data (see the “Material and methods” section). For each assembly, we computed the following metrics: the number of misassemblies, ortholog completeness, and cumulative length/Nx distributions (Table 1). These assessments were carried out using BUSCO [29] for ortholog completeness (Additional file 1: Fig. S1) and QUAST-LG’s validation pipeline [30] to search for misassemblies introduced in the scaffolds. QUAST-LG is an updated version of the traditional QUAST pipeline specifically designed for large genomes and is a state-of-the-art software for assembly evaluation and comparison. We used QUAST to verify that annotations transferred successfully from the reference v1 assembly to the instaGRAAL v4 assembly and that no structural discrepancy (a.k.a. misassemblies) was found in the instaGRAAL v4 assembly with respect to the reference v1 assembly. We followed the terminology used by both programs, such as the BUSCO definition of ortholog and completeness, as well as QUAST’s classification system of contig and scaffold misassemblies.

The corrected instaGRAAL assembly was of better quality than both the LG v2 and GRAAL v3 assemblies (Table 1 and Additional file 1: Fig. S2). The corrected assembly incorporated 795 of the v1 genome scaffolds (96.8% of the sequence data) into the 27 chromosomes based on the high-density genetic map [21], compared to 531 for the LG v2 assembly (90.5% of the sequence data). Moreover, this assembly contained fewer misassemblies and was more complete in terms of BUSCO ortholog content. For some metrics, the differences were marginal, but always in favor of the corrected instaGRAAL v4 assembly. BUSCO completeness was similar (76.2%, 76.9%, and 77.6% for the GRAAL v3 assembly, LG v2, and corrected instaGRAAL v4 assemblies, respectively) (Additional file 1: Fig. S2) and an improvement over the 75.9% of the v1 assembly. These absolute numbers remain quite low, presumably because of the lack of a set of orthologs well adapted to brown algae.

All quantitative metrics, such as N50, L50, and cumulative length distribution, increased dramatically when compared with the reference genome v1 assembly (Table 1). N50 increased more than tenfold, from 496,777 bp to 6,867,074 bp after the initial scaffolding and to 6,942,903 bp after the correction steps. 99.4% of the sequences in the 1018 contigs were integrated into the 27 largest scaffolds after instaGRAAL processing. Overall, the analysis indicated that many of the rearrangements found in the LG v2 assembly were potentially errors and that both GRAAL and instaGRAAL were efficient at placing large regions where they belong in the genome, albeit less accurately for GRAAL and in the absence of correction. These statistics underline the importance of the post-scaffolding correction steps and the usefulness of a program that automates these steps.

### Comparison between the *Ectocarpus* sp. instaGRAAL and linkage group assemblies

Compared to the LG v2 assembly, the corrected instaGRAAL v4 assembly lost 23 scaffolds but gained 287 that the genetic map had been unable to anchor to chromosomes (Additional file 1: Table S2). We observed few conflicts between the two assemblies, and the linkage markers are globally consistent with the instaGRAAL scaffolds (Additional file 1: Fig. S3). One major difference is that instaGRAAL was able to link the 4th and 28th linkage groups (LG) that were considered to be separate by the genetic map [26] because of the limited number of recombination events observed. The fusion in the instaGRAAL v4 assembly is consistent with the fact that the 28th LG is the smallest, with only 54 markers over 41.8 cM and covering 3.8 Mb. The 28th LG has a very large gap which might reflect uncertainty in the ordering of the markers. Interestingly, this gap is located at one end of the group, precisely where instaGRAAL now detects a fusion with the 4th LG. In addition, the fact that there is no mix between the 4th and 28th LGs on the merged instaGRAAL (pseudo) chromosome but rather a simple concatenation suggests that the genetic map was unsuccessful in joining those two LGs, but that instaGRAAL correctly assembled the two LGs (see Additional file 1: Table S3 for correspondences between LGs and instaGRAAL super scaffolds).

instaGRAAL was also more accurate than the genetic map in orienting scaffolds (Additional file 1: Table S2). Among the scaffolds that were oriented in the LG v2 assembly, about half of the “plus” orientated were actually “minus” and vice versa. The limited number of markers detected in the scaffolds anchored to the genetic map was likely the reason for this high level of incorrect orientations.

### Scaffolding of the *Desmarestia herbacea* genome

To test and validate instaGRAAL on a second, larger genome, we generated an assembly of the haploid genome of *D. herbacea*, a brown alga that had not been sequenced before. We set up the assembly pipeline and subsequent scaffolding from raw sequencing reads to assess the robustness of instaGRAAL with de novo, non-curated data. The pipeline proceeded as follows: first, we acquired 259,556,174 short paired-end shotgun reads (Illumina HiSeq2500 and 4000) as well as 1,353,202 long reads generated using PacBio and Nanopore (about 150× short reads and 15× long reads). Sequencing reads were processed using the hybrid MaSuRCA assembler (v3.2.9) [31], yielding 7743 contigs representing 496 Mb (Table S4). We generated Hi-C data following a protocol similar to that used for *Ectocarpus* sp. (see the “Material and methods” section). Briefly, 101,879,083 reads were mapped onto the hybrid assembly, yielding 7,649,550 contacts linking 1,359,057 fragments. We then ran instaGRAAL using similar default parameters to that used for *Ectocarpus* sp., for the same number of cycles. We corrected the resulting scaffolds. The scaffolding process resulted in 40 scaffolds larger than 1 Mb (Additional file 1: Fig. S4, S5, S6), representing 98.1% of the initial, filtered scaffolding and 89.3% of the total initial genome after correction and reintegration. The exact number of chromosomes in *D. herbacea* is unknown but was estimated to be ~ 23, and possibly up to 29, based on cytological observations [32]. Most (35) of the scaffolds generated by instaGRAAL were syntenic with the 27 *Ectocarpus* sp. scaffolds. Among the remaining five scaffolds, one corresponded to the genome of an associated bacterium, and two to large regions with highly divergent GC content (37 and 40% vs. 48% for the rest of the genome) and no predicted *D. herbacea* genes. Overall, instaGRAAL successfully scaffolded the *D. herbacea * genome, although the final number of scaffolds remained slightly higher than the estimated number of chromosomes in this species.

### Comparisons with existing methods

To date, only a limited number of Hi-C-based scaffolding programs are publicly available, and as far as we can tell, no detailed comparison has been performed between the existing programs to assess their respective qualities and drawbacks. In an attempt to benchmark instaGRAAL, we ran SALSA2 [33] and 3D-DNA on the same *Ectocarpus* sp. v1 and *Desmarestia herbacea* reference genome and Hi-C reads. 3D-DNA is a scaffolder that was hallmarked with the assembly of *Aedes aegypti*, and SALSA2 is a recent program with a promising approach that directly integrates Hi-C weights into the assembly graph. For *Ectocarpus* sp., SALSA2 ran for nine iterations and yielded 1042 scaffolds, with an N50 of 6,552,506 (L50 = 11). Its BUSCO completeness was 77.6%, a level identical to that obtained with instaGRAAL. Overall, the metrics were satisfactory but SALSA2 was outperformed by instaGRAAL post-correction. The contact map of the resulting SALSA2 assembly displayed noticeably unfinished scaffolds (Additional file 1 Fig. S7 and S8). This, coupled with a lower N50 value, suggests that instaGRAAL is more successful at merging scaffolds when appropriate.

We computed similar size and completeness statistics for the final instaGRAAL *D. herbacea* assembly and compared these to the values obtained with SALSA2 and 3D-DNA. We also mapped the Hi-C reads onto all three final assemblies in order to qualitatively assess the chromosome structure. The results are summarized in Table S4.

Briefly, statistics across assemblies were similar; the corrected instaGRAAL assembly had 73% BUSCO completeness, consistent with the values of 73.6% and 70.3% obtained for SALSA2 and 3D-DNA, respectively. However, the Lx/Nx metrics diverged significantly; the instaGRAAL assembly N50 was 12.4 Mb, similar to SALSA2 (12.8) and much larger than 3D-DNA (0.2 Mb). However, visual inspection of the contact maps indicated that neither SALSA2 nor 3D-DNA succeeded in fully scaffolding the genome of *Desmarestia herbacea* (Additional file 1: Fig. S7). Notably, SALSA2 created a number of poorly supported junctions to generate chromosomes, whereas 3D-DNA failed to converge towards any kind of structure. In contrast, although the instaGRAAL final assembly still contains input contigs that are incorrectly positioned, a coherent structure corresponding to 40 scaffolds (including contaminants) emerged (Additional file 1: Fig. S4). One possibility is that the de novo MaSuRCA assembly was low quality, likely due to the low coverage of long reads, which would have resulted in alignment errors that disrupted the contact distribution and subsequent Hi-C scaffolding. Another possible explanation for these differences is that it remains difficult to dissect all the options and tunable parameters of these scaffolders, and therefore that we did not find the optimal combination with respect to the *D. herbacea* draft assembly. Nevertheless, these results highlight the robustness of instaGRAAL which was able to scaffold the *D. herbacea* genome using default parameters.

### Scaffolding the human genome

To confirm that instaGRAAL scaffolds larger (Gb scale) genomes in a reasonable time, we ran it on the GRCh38 human genome sliced into 300-kb segments (artificial assembly), using a Hi-C dataset generated with an Arima Genomics Hi-C kit (see the “Material and methods” section). instaGRAAL was run for 15 cycles, with the parameter --levels sets to 5, and the scaffolds were subsequently corrected with instaGRAAL-polish. We obtained a total of 1302 scaffolds, out of which 24 have a length ranging from 18 to 239 Mb. These 24 chromosome-level scaffolds are represented in the contact map in Additional file 1: Fig. S9. These scaffolds have an N50 and an NGA50 of 143 Mb, close to the 145 Mb obtained for the reference genome (Table 2; the results from [33] using SALSA2 are included). The dot plot similarity map between the instaGRAAL scaffolds and reference genome assembly (Additional file 1: Fig. S10) shows that the 22 autosomes and the X chromosome were recovered by instaGRAAL (although a few relocations and inversions remain visible). In addition, a 24th scaffold is visible composed of sequences also in contacts with the other scaffolds, corresponding to repeated sequences clustering together. instaGRAAL produced scaffolds with a lower contiguity than those of SALSA2: while their N50 are comparable, the N75 of instaGRAAL is significantly lower. However, the number of complete genomic features in the instaGRAAL scaffolds is largely improved compared to the input fragments, while SALSA2 only slightly increased this score. These results suggest that although the scaffolds of instaGRAAL are less contiguous, they are of better quality. Since these scaffolds were obtained after only 15 cycles, increasing the number of cycles is very likely to improve the N75. All in all, and though additional work is needed to polish such an output as with all assembly projects, these results confirm that instaGRAAL can efficiently scaffold large genomes.

**Table 2:** Comparison of Nx, NGx (i.e., Nx with respect to the original human reference genome assembly; in bp), and other QUAST statistics for the different assemblies (artificial assembly, corrected instaGRAAL, and SALSA2) of the *Homo sapiens* genome

	Reference genome assembly	Artificial assembly	instaGRAAL	SALSA2
N50	145,138,636	300,000	143,373,745	152,389,473
NG50	145,138,636	300,000	143,373,745	152,389,473
N75	107,043,718	300,000	89,477,166	130,103,422
NG75	107,043,718	300,000	82,128,910	103,672,000
L50	9	5165	9	9
LG50	9	5454	9	9
L75	15	7747	15	15
LG75	15	8181	17	17
No. of genomic features	3,625,295 + 305 part	3,411,473 + 44,299 part	3,456,227 + 3836 part	3,415,115 + 44,127 part
Genome fraction (%)	100.0	94.6	94.6	94.5
No. of misassemblies	9	0	776	438

### Benchmarking of the system requirements

To quantify the improvements made over the original GRAAL program, we ran both GRAAL and instaGRAAL over the *Ectocarpus* sp. v1 genome separately and measured the peak memory load, the graphics card memory load taken by the contact maps, and the per-cycle runtime as averaged from 20 cycles. The results are summarized in Table S5. As expected, the memory load on the graphics card is an order of magnitude smaller for instaGRAAL, while the peak RAM and runtime are several times smaller. The shrinkage of memory requirements is predicted by the use of sparse data structures and the fact that our original dataset for *Ectocarpus* sp. is relatively lean when compared to the size of the genome. The origin of the accelerated runtime is less clear and could be due to multiple contributions to the program, including the use of sparse data structures but also external contributions (e.g., porting to Python 3, upgraded libraries, or more recent CUDA versions).

It is important to note, however, that these results are highly specific to the hardware and data used here, and due to the many different factors involved, any comparison should stick to orders of magnitude. Nevertheless, this confirms that instaGRAAL’s improvements over GRAAL are very substantial and make it suitable for modern, large genome assembly projects.

## Discussion

instaGRAAL is a Hi-C scaffolding program that can process large eukaryotic genomes. Below, we discuss the improvements made to the program, its remaining limitations, and the steps that will be needed to tackle them.

### Refinement/correction step

An important improvement of instaGRAAL compared to GRAAL relates to post-scaffolding corrections. Local misassemblies, e.g., local bin inversions or disruptive insertions of small scaffolds within larger ones, are an inevitable consequence of the algorithm’s most erratic random walks. These small misassemblies are retained because flipping a bin does not markedly change the relative distance of an RFs relative to its neighbors, and because small scaffolds typically carry less signal and therefore exhibit a greater variance in terms of acceptable positions. Depending on the trust put in the initial set of contigs, one may be unwilling to tolerate these changes as well as “partial translocations,” i.e., the splitting of an original contig into two scaffolds. The prevalence of such mistakes can be estimated by comparing the orientation of bins relative to their neighbors in the instaGRAAL v4 assembly vs. the original assembly (v1 assembly). Our assumption is that if a single bin was flipped or split by instaGRAAL, this was likely a mistake that needed to be corrected. Consequently, we chose to remain faithful to the input contigs of the original v1 assembly, given that the initial *Ectocarpus sp.* v1 (reference) genome sequence was based on Sanger reads. Our correction therefore aims at reinstalling the initial contig structure and orientation while preserving to a maximum extent the overall instaGRAAL scaffold structure.

In addition, our correction reintegrates into the assembly the bins removed during the initial filtering process according to their position along the original assembly contigs. Most filtered bins corresponded to the extremities of the original contigs, because their size depended on the position of the restriction sites within the contig, or because they consisted of repeated sequences with little or no read coverage. The tail filtering correction step inserts these bins back at the extremities of these contigs in the instaGRAAL assembly.

The combination of a probabilistic algorithm with a deterministic correction step provides robustness to instaGRAAL. First, the MCMC step identifies, with few prior assumptions, a high-likelihood family of genome structures, almost always very close to the correct global scaffolding. The correction step combines this result with prior assumptions made about the initial contig structures generated through robust, established assembly programs, refining the genomic structure within each scaffold. To give the user a fine-grained degree of control over our correction procedures, the implementation into instaGRAAL is split into independent modules that each assume about the initial contig structure necessary to perform the correction: the “reorient” module assumes that the initial contigs do not display inversions, and the “rearrange” module assumes that there are no relocations within contigs.

We underline that despite the improvements brought about by these new procedures, instaGRAAL assemblies remain perfectible, notably because of the reliance on the quality of the input contigs used for correction. For instance, the *D. herbacea* genome heavily relies on contigs generated from a de novo hybrid assembly, and the contact maps in Additional file 1: Fig. S4, S5, and S6 show some extraneous signal that may point at misassemblies. Analogous observations may be made with respect to *Ectocarpus* sp. in Additional file 1: Fig. S11. In addition, inherent limits to Hi-C technology such as the restriction fragment size mean that there are going to be false junctions between fragments or bins. This is only a problem if one chooses not to reconstruct every input contig within a newly formed scaffold with our correction procedure, i.e., one is distrustful of the initial input contigs. This was not the case for *Ectocarpus* sp. but could be argued for *D. herbacea*, where the de novo contigs generated from 15× coverage may be of poor quality.

### Sparse data handling

The implementation of a sparse data storage method in instaGRAAL allows much more intense computation than with GRAAL. Because the majority of map regions are devoid of contacts, instaGRAAL essentially halves the order of magnitude of both algorithm complexity and memory load, i.e., they increase roughly linearly with the size of the genome instead of geometrically. This improvement potentially allows the assembly of Gb-sized genomes in 4 to 5 days using a laptop (i.e., much faster with more computational resources).

### Filtering

Variations in GC% along the genome, and/or other genomic features, can lead to variation in Hi-C read coverage and impair interpretation of the Hi-C data. Correction and attenuation procedures that alleviate these biases are therefore commonly used in Hi-C studies [34–36]. However, these procedures are not compatible with instaGRAAL’s estimation of the contact distribution (for more details, see [37]). A subset of bins will therefore diverge strongly from the others, displaying little if no coverage. A filtering step is needed to remove these bins as they would otherwise impact the contact distribution and the model parameter estimation. These disruptive bins represent a negligible fraction of the total genome (< 3% of the total genome size of Ectocarpus sp., for instance) and are reincorporated into the assembly during correction. On the other hand, a subset of bins representing small, individual scaffolds are not reinserted during correction and are added to the final assembly as extra-scaffolds (as in all sequencing projects). Additional analyses and new techniques such as long or linked reads are needed to improve the integration of these scaffolds into the genome.

### Resolution

The binning procedure will influence the structure of the final assembly as well as its quality. For example, low-level binning (e.g., one bin = three RFs) will lead to an increased number of bins and a large, sparse contact map with a low signal-to-noise ratio, where many of the bins display poor read coverage as on average they will have fewer contacts with their immediate neighbors. Because of the resulting low signal-to-noise ratio, an invalid prior model will be generated, and when referring to this model, the algorithm will fail to scaffold the bins properly, if at all. Moreover, due to its probabilistic nature, the algorithm will generate a number of false positive structural modifications such as erroneous local inversions or permutations of bins. The numerous bins will create more genome structures to explore to handle all the potential combinations, and exploring this space until convergence will take longer and be computationally demanding.

On the other hand, one of the advantages of instaGRAAL is its ability to scaffold fragments or bins instead of contigs themselves. This has two main effects: First, it dodges the size bias issue whereby larger contigs will feature more contacts and will need to be normalized. Second, it allows for greater flexibility when exploring genome space, potentially uncovering misassemblies within input contigs. This is more relevant in the case of large contigs generated with long reads. And even if we assume that the initial contigs are completely devoid of misassemblies, this flexibility is useful when the contact distribution is disrupted by extraneous signals and the scaffolder needs to decide between two regions of similar affinity. The correction tool subsequently reconstructs the initial contigs from these rough arrangements, as discussed above (reference-based correction).

An optimal resolution is therefore a compromise between the bin size, the coverage, and the quality of the input contigs from the original assembly. Although a machine powerful enough operating on an extremely contact-rich matrix would be successful at any level, it is unclear whether such resources are necessary. Our present assemblies (e.g., 1 bin = 81 RFs for both; see the “Material and methods” section) had good quality metrics after a day’s worth of calculation on a standard desktop computer for *Ectocarpus* sp. and *D. herbacea*. Moreover, convergence was qualitatively obvious after a few cycles. This suggests that more computational power yields diminishing returns and therefore that appropriate correction procedures are a more efficient approach for remaining misassemblies.

### Binning

The fragmentation of the original assembly used to generate the initial contact map has a substantial effect on the quality of the final scaffolding. Because binning cannot be performed beyond the resolution of individual input contigs, however small they may be, there is a fixed upper limit to the scale at which a given matrix can be binned. A highly fragmented genome with many small input contigs will necessarily generate a high-noise, high-resolution matrix. Attempts to reassemble a genome based on such a matrix will run into the problems discussed above (resolution). This limitation can be alleviated, to some extent, by discarding the smallest contigs, with the hope that the remaining contigs will cover enough of the genome. The input contigs that are removed can be reintegrated into the final scaffold during the correction steps. This ensures an improved Nx metric while retaining genome completeness. It should be noted, however, that the size of the input contigs is important as they need to contain sufficient restriction sites, and each of the restriction fragments must have sufficient coverage. The choice of enzyme and the frequency of its corresponding site are thus crucial. For instance, with an average of one restriction site every 600 to 1000 bp for *Dpn*II, input contigs as short as 10 kb may contain enough information to be correctly reassembled. The restriction map therefore strongly influences both the minimum limit on N50 and genome fragmentation.

### Benchmarking

In order to test our tool against existing programs, we ran two scaffolders available online (SALSA2 and 3D-DNA) on our two genomic datasets. In all instances, instaGRAAL proved more successful at scaffolding both genomes. However, we have not extensively tested all the combinations of parameters of both programs, and acknowledge the difficulty in designing and implementing Hi-C scaffolding pipelines with extensive dependencies that compound the initial complexity of the task and add yet more configurable options to know in advance. Finding the correct combination of CUDA and Python dependencies to install instaGRAAL on a given machine can be challenging as well. Therefore, our benchmarking attempt should be rather seen as a way to stress the importance of implementing sensible default parameters that readily cover as many use cases as possible for the end user. There is almost no doubt that both 3D-DNA and SALSA2, with the appropriate parameters and correction steps, would produce satisfying scaffolding; on the other hand, knowing which input parameters has to be specified in advance is a non-trivial task, especially given the computational resources needed for a single scaffolding run. With instaGRAAL, we wish to combine the simplicity of a default configuration that works in most instances, with the flexibility offered by the power of MCMC methods.

### Choosing your parameters

In the benchmarking, we have discussed why some parameters are crucial and why we took care, through trial-and-error, to implement sensible defaults for future similar assembly projects. On the other hand, it is crucial that such defaults be not the result of overfitting for the assemblies we tested. However, none of what we outlined previously assumes anything specific about the genomes at hand beyond very broad metrics such as their total size or N50. The parameters of the program scale intuitively with such metrics. For larger genomes, one may simply increase the size of the bins so that the contact map does not grow too large, which is what we did for the human genome. The N50 sets the resolution limit in that it is often desirable to be able to break down contigs into many bins of roughly equal size so as not to run into the aforementioned size bias and also to be able to give more flexibility to the program. For instance, an N50 close to 100 kb should not feature bins larger than 50–60 kb. Oftentimes, however, such minutiae is not necessary, and for most genome projects ranging across 10^7^–10^9^ bp, instaGRAAL will typically work out of the box with default parameters. For instance, we kept the same parameters for both algae and only switched to a lower resolution (higher bin size) for the human genome to scale with its size. When needed, through these simple rules of thumb, one may adapt the defaults to other genomes with more extreme metrics.

### Handling diploid genomes

As assembly projects have grown more complex and exhaustive, expectations have increased as well. Assembling diploid, if not polyploid, genomes with well-characterized haplotypes is a stumbling block in the field. Moreover, such problems are more likely to be encountered as the low-hanging fruit gets picked. Typical projects involve assembling many individual complete human genomes with haplotypes, or the sequencing and scaffolding of even larger and more complex genomes such as that of plants. In this context, instaGRAAL in particular (and Hi-C in general) is relatively agnostic, as its success or failure will hinge on the reference genome being properly haplotyped in the first place. While it may prove intractable to phase haplotypes directly from only Hi-C data, instaGRAAL will conserve such information when provided in the first place. This is because the scaffolder is robust to local disruptions like haplotype-induced mapping artifacts. It has been shown that GRAAL and by extension instaGRAAL will eventually resolve such disruptions even when the distribution is noisy, as long as the general three-parameter model (and power law) still holds globally [13, 19, 20]. In other words, even though instaGRAAL cannot “guess” whether a given reference sequence is homologous or heterozygous without considerable difficulty, it can still cleanly scaffold chromosome pairs from clear contig pairs because the global 3D intra-signature from a given contig is too strong to be confused with mapping artifacts in a pair. Should such information be missing, the scaffolder will likely interlace all regions into a giant linkage group. In that respect, instaGRAAL could interface well with diploid classical assemblers and is suitable for any pipeline integration involving diploid genomes. More work is needed in that direction so that the scaffolder does not rely that strongly on the quality of the input contigs to work out haplotypes.

### Integrating information from the Hi-C analysis with other types of data

Aggregating data from multiple sources to construct a high-quality genome sequence remains a challenging problem with no systematic solution. As long-read technologies become more affordable, there is an increasing demand to reconcile the scaffolding capabilities of Hi-C-based methods with the ability of long reads to span regions that are difficult to assemble, such as repeated sequences. The most intuitive approach would be to perform Hi-C scaffolding on an assembly derived from high-coverage and corrected long reads, as was done for several previous assembly projects [16, 38]. Alternative approaches also exist, such as generating Hi-C- and long-read-based assemblies separately and merging them using programs such as CAMSA [39] or Metassembler [40]. Pipelines such as PBJelly [41] have proven successful at filling existing gaps in draft genomes, regardless of their origin, with the help of long reads. Lastly, with assembly projects involving both long and short reads, hybrid assemblies and hybrid polishing have become an important focus. Polishers such as Racon [42] or Pilon [43] are widely used, and new tools such as HyPo are also emerging [44]. Yet the question of which kind of pipeline to use (e.g., Racon to Hi-C scaffolding to Pilon, or Racon to Pilon to Hi-C scaffolding, etc.) along which hybrid assembler (Masurca, Alpaca, hybridSPAdes, etc.) [31, 45, 46] can prove cumbersome, and often finding the process yielding the most satisfying output in terms of metrics involves much trial-and-error with different configurations. InstaGRAAL shows that high-quality metrics can still be attained without the help of long reads, but long-read polishing may still be necessary in order to get rid of the lingering errors we mentioned. Long reads are not the only type of data that can be used to improve assemblies. Linkage maps, RNA-seq, optical mapping, and 10X technology all provide independent data sources that can help improve genome structure and polish specific regions. The success of future assembly projects will hinge on the ability to process these various types of data in a seamless and efficient manner.

## Material and methods

### Preparation of the Hi-C libraries

The Hi-C library construction protocol was adapted from [8, 47]. Briefly, partheno-sporophyte material was chemically cross-linked for 1 h at RT using formaldehyde (final concentration, 3% in 1× PBS; final volume, 30 ml; Sigma-Aldrich, St. Louis, MO). The formaldehyde was then quenched for 20 min at RT by adding 10 ml of 2.5 M glycine. The cells were recovered by centrifugation and stored at − 80 °C until use. The Hi-C library was then prepared as follows. Cells were resuspended in 1.2 ml of 1× *Dpn*II buffer (NEB, Ipswich, MA), transferred to a VK05 tubes (Precellys, Bertin Technologies, Rockville, MD), and disrupted using the Precellys apparatus and the following program ([20 s—6000 rpm, 30 s—pause] 9× cycles). The lysate was recovered (around 1.2 ml) and transferred to two 1.5-ml tubes. SDS was added to a final concentration of 0.3%, and the 2 reactions were incubated at 65 °C for 20 min followed by an incubation of 30 min at 37 °C. A volume of 50 μl of 20% Triton-X100 was added to each tube, and incubation was continued for 30 min. *Dpn*II restriction enzyme (150 units) was added to each tube, and the reactions were incubated overnight at 37 °C. Next morning, reactions were centrifuged at 16,000×*g* for 20 min. The supernatants were discarded, and the pellets were resuspended in 200 μl of NE2 1× buffer and pooled (final volume = 400 μl). DNA extremities were labeled with biotin using the following mix (50 μl NE2 10× buffer, 37.5 μl 0.4 mM dCTP-14-biotin, 4.5 μl 10 mM dATP-dGTP-dTTP mix, 10 μl Klenow 5 U/μl) and an incubation of 45 min at 37 °C. The labeling reaction was then split in two for the ligation reaction (ligation buffer—1.6 ml, ATP 100 mM—160 μl, BSA 10 mg/ml—160 μl, ligase 5 U/μl—50 μl, H2O—13.8 ml). The ligation reactions were incubated for 4 h at 16 °C. After addition of 200 μl of 10% SDS, 200 μl of 500 mM EDTA, and 200 μl of proteinase K 20 mg/ml, the tubes were incubated overnight at 65 °C. DNA was then extracted, purified, and processed for sequencing as previously described (Lazar-Stefanita et al. [47]). Hi-C libraries were sequenced on a NextSeq 550 apparatus (2 × 75 bp, paired-end Illumina NextSeq with the first ten bases acting as barcodes; Marbouty et al. [15]).

### Contact map generation

Contact maps were generated from reads using the hicstuff pipeline for processing generic 3C data, available at https://github.com/koszullab/hicstuff. The backend uses the bowtie2 (version 2.2.5) aligner run in paired-end mode (with the following options: --maxins 5 –very-sensitive-local). Alignments with mapping quality lower than 30 were discarded. The output was in the form of a sparse matrix where each fragment of every chromosome was given a unique identifier and every pair of fragments was given a contact count if it was non-zero.

Fragments were then filtered based on their size and total coverage. First, fragments shorter than 50 bp were discarded. Then, fragments whose coverage was less than one standard deviation below the mean of the global coverage distribution were removed from the initial contact map. A total of 6,974,350 bp of sequences was removed this way. An initial contact distribution based on a simplified a polymer model [27] with three parameters was first computed for this matrix. Finally, the instaGRAAL algorithm was run using the resulting matrix and distribution.

For the *Ectocarpus* sp. genome, instaGRAAL was run at levels 4 (*n* = 81 RFs), 5 (*n* = 243 RFs), and 6 (*n* = 729 RFs). Levels 5 and 6 were only used to check for genome stability and consistency in the final chromosome count. Level 4 was used for all subsequent analyses. All runs were performed for 250 cycles. The starting fragments for the analysis were the reference genome scaffolds split into restriction fragments. The same parameters were used for the *D. herbacea* genome. The same parameters were used for the human genome, except we used level 6 instead of 4.

### Correcting genome assemblies

The assembled genome generated by instaGRAAL was corrected for misassemblies using a number of simple procedures that aimed to reinstate the local structure of the input contigs of the original assembly where possible. Briefly, bins belonging to the same input contig were juxtaposed in the same relative positions as in the original assembly. Small groups of bins were preferentially moved to the location of larger groups when several such groups were present in the assembly. The orientations of sets of bins that had been regrouped in this manner were modified so that orientation was consistent and matched that of the majority of the group, re-orientating minority bins when necessary. Both steps are illustrated in Fig. 4. Finally, fragments that had been removed during the filtering steps were reincorporated if they had been adjacent to an already integrated bin in the original assembly. The remaining sequences that could not be reintegrated this way were appended as non-integrated scaffolds.

### Validation metrics

Original and other assembly metrics (Nx, GC distribution) were obtained using QUAST-LG [30]. Misassemblies were quantified using QUAST-LG with the minimap2 aligner in the backend. Ortholog completeness was computed with BUSCO (v3) [29]. Assembly completeness was also assessed with BUSCO. The evolution of genome metrics between cycles was obtained using instaGRAAL’s own implementation.

### Validation with the genetic map

The validation procedure with respect to linkage data was implemented as part of instaGRAAL. Briefly, the script considers a set of linkage group where regions are separated by SNP markers and a set of Hi-C scaffolds where regions are bins separated by restriction sites. It then finds best-matching pairs of linkage groups/scaffolds by counting how many of these regions overlap from one set to the other. Then, for each pair, the bins in the Hi-C scaffold are rearranged so that their order is consistent with that of the corresponding linkage group. Such rearrangements are parsimonious and try to alter as little as possible. Since there is not a one-to-one mapping from restriction sites to SNP markers, some regions in the Hi-C scaffolds are not present in the linkage groups, in which case they are left unchanged. When the Hi-C scaffolds are altered this way, as was found in the case of the raw GRAAL v3 assembly, the script acts as a correction. When the scaffolds are unchanged, as was the case with the instaGRAAL corrected v4 assembly, the script acts as a validation.

### Benchmarking with other assemblers

For each genome, the 3D-DNA program was run using the run-assembly-pipeline.sh entry point script with the following options: -i 1000 --polisher-input-size 10000 --splitter-input-size 10000. The Hi-C data was prepared with the Juicer pipeline as recommended by 3D-DNA’s documentation. The SALSA2 program was run with the –cutoff=0 option, and misassembly correction with the –clean=yes option. No expected genome size was provided. The program halted after 9 iterations for *Ectocarpus* sp. and 18 iterations for *D. herbacea*. Hi-C data was prepared with the Arima pipeline as recommended by SALSA2’s documentation. The similarity dot plot between corrected instaGRAAL and SALSA scaffolds was generated with minimap2.

### Benchmarking with the human genome

We followed a procedure similar to the benchmark analysis detailed in [33]*.* Briefly, the GRCh38 reference genome was cut into 300-kb fragments. The Hi-C library generated using an Arima Genomics kit was aligned against the genome (SRA: SRR6675327). instaGRAAL was run on the resulting contact map, using the same default parameters as for the algae genomes, except we increased the resolution level to 6 (from 4). The similarity dot plot between instaGRAAL and SALSA scaffolds was generated with minimap2, with the options -DP -k19 -w19 -m200.

### Software tool requirements

The instaGRAAL software is written in Python 3 and uses CUDA for the computationally intensive parts. It requires a working installation of CUDA with the pycuda library. CUDA is a proprietary parallel computing framework developed by NVIDIA and requires a NVIDIA graphics card. The scaffolder also requires a number of common scientific Python libraries specified in its documentation. The instaGRAAL website lists computer systems onto which the program was successfully installed and run.

## Supplementary information

**Additional file 1.** Supplementary tables and figures.

**Additional file 2.** Review history.

## Data Availability

The datasets generated and analyzed in the present work are available in the SRA repository, SRR8550777 [48]. The instaGRAAL software and its documentation are freely available under the GPL-3.0 license at https://github.com/koszullab/instaGRAAL [49]. Assemblies, contact maps, and relevant materials for the reproduction of the main results and figures are available at https://github.com/koszullab/ectocarpus_scripts [50].
