# Supplementary material for: instaGRAAL: chromosome-level quality scaffolding of genomes using a proximity ligation-based scaffolder
Source: Genome Biol. 2020 Jun 18;21:148. doi: 10.1186/s13059-020-02041-z (PMC7386250; doi:10.1186/s13059-020-02041-z)
Supplement: Supplementary file 1 — Additional file 1. Supplementary tables and figures. [file 13059_2020_2041_MOESM1_ESM.docx]

**Supplementary file**

**Chromosome-level quality scaffolding of brown algal genomes usingInstaGRAAL, a proximity ligation-based scaffolder**

Lyam Baudry^1,2^, Nadège Guiglielmoni^1,6^, Hervé Marie-Nelly^1,2^, Alexandre Cormier^4^, Martial Marbouty^1^, Komlan Avia^4*^, Yann Loe Mie^3^, Olivier Godfroy^4^, Lieven Sterck^4^, J. Mark Cock^4^, Christophe Zimmer^5^, Susana M. Coelho^4,&^, Romain Koszul^1,&^

**Table of contents**

**Table S1:** example of a sparse matrix.

**Table S2:** comparison of the integrated sequences between the different assemblies and the reference v1 genome assembly for *Ectocarpus* sp.

**Table S3:** correspondences between instaGRAAL super scaffolds and linkage groups for the *Ectocarpus* sp. genome.

**Table S4:** metrics of *Desmarestia herbacea* scaffolding using three different programs.

**Table S5:** performance of GRAAL and instaGRAAL at scaffolding the *Ectocarpus* sp. genome.

**Figure S1:** normalized contact map of *Ectocarpus* chromosomes 1 to 27.

**Figure S2**: estimates of BUSCO-completeness for the three *Ectocarpus* sp. assemblies and the reference genome.

**Figure S3:** Linkage markers *vs.* scaffold positions for all linkage groups/chromosomes

**Figure S4:** The 40 main scaffolds of *Desmarestia herbacea* after instaGRAAL scaffolding.

**Figure S5:** Contact maps of the first twenty newly formed scaffolds/putative chromosomes of *Desmarestia herbacea*

**Figure S6:** The last twenty newly formed scaffolds/putative chromosomes of Desmarestia herbacea.

**Figure S7**: Comparisons of contact maps for three different scaffolders across two species.

**Figure S8**: Similarity dotplot of the SALSA2 vs. instaGRAAL 27 scaffolds for *Ectocarpus* sp*.*

**Figure S9:** Contact map of the *Homo sapiens* genome, fragmented in 300 kb sequences, after scaffolding with instaGRAAL

**Figure S10**: Similarity dotplot of the instaGRAAL *vs.* reference scaffolds for the GRCh38 human genome.

**Figure S11:** All 27 newly formed scaffolds/putative chromosomes of *Ectocarpus* sp.

**Movie S1**: the iterative scaffolding process can be visualized on a movie accessible through the following link.

<https://github.com/koszullab/ectocarpus_scripts/blob/master/images/matrix_evolution.gif>

Each frame corresponds to a cycle during which each fragment has been processed once.

| id_frag_a | id_frag_b | n_contact |
| --- | --- | --- |
| 0 | 0 | 1368 |
| 0 | 1 | 21 |
| 0 | 2 | 7 |
| 0 | 3 | 3 |
| 0 | 4 | 5 |
| 0 | 7 | 5 |
| 0 | 8 | 1 |
| 0 | 9 | 1 |
| 0 | 12 | 2 |
| 0 | 15 | 1 |
| 0 | 22 | 1 |
| 0 | 23 | 1 |
| 0 | 26 | 1 |
| 0 | 27 | 1 |
| 0 | 33 | 2 |
| 0 | 36 | 2 |
| 0 | 37 | 1 |
| 0 | 51 | 1 |
| 0 | 69 | 1 |
| 0 | 74 | 2 |
| 0 | 76 | 1 |
| 0 | 97 | 1 |
| 0 | 99 | 1 |
| 0 | 107 | 1 |

**Table S1:** example of a sparse matrix.

|  | v1 genome assembly | linkage group v2 assembly | Corrected instaGRAAL v4 assembly |
| --- | --- | --- | --- |
| Scaffolds integrated into linkage groups (out of 1561) | 325 | 531 | 793 |
| Percent sequence data Integrated into linkage groups | 70.10 % | 90.50 % | 96.80 % |
| Integrated oriented scafflolds in the linkage groups | 12 % | 49 % | 100 % |
| Number of linkage groups | 34 | 28 | 27 |

**Table S2:** comparison of the integrated sequences between the different assemblies and the v1 assembly for *Ectocarpus* sp.

| instaGRAAL v4 assembly | Linkage group v2 assembly |
| --- | --- |
| 1 | 1 |
| 2 | 21 |
| 3 | 4 and 28 |
| 4 | 5 |
| 5 | 13 |
| 6 | 6 |
| 7 | 12 |
| 8 | 7 |
| 9 | 27 |
| 10 | 26 |
| 11 | 3 |
| 12 | 2 |
| 13 | 8 |
| 14 | 14 |
| 15 | 10 |
| 16 | 11 |
| 17 | 19 |
| 18 | 16 |
| 19 | 9 |
| 20 | 15 |
| 21 | 18 |
| 22 | 20 |
| 23 | 24 |
| 24 | 23 |
| 25 | 17 |
| 26 | 25 |
| 27 | 22 |

**Table S3:** correspondences between instaGRAAL super scaffolds and linkage groups from the v2 assembly for the *Ectocarpus* sp. genome.

|  | ***De novo* original assembly** | **3D-DNA** | **SALSA2** | **instaGRAAL** |
| --- | --- | --- | --- | --- |
| N50 (bp) | 184,092 | 175,000 | 12,780,148 | 12,444,485 |
| L50 | 697 | 545 | 11 | 17 |
| contig count | 7,743 | 5385 | 4,827 | 4,304 |
| BUSCO completeness | 72.6 | 70.7 | 73.6 | 73 |

**Table S4:** metrics of *Desmarestia herbacea*assemblies using three different programs.

|  | **GRAAL** | **instaGRAAL** |
| --- | --- | --- |
| Peak memory load (Gb) | 2.5 | 1.1 |
| Memory used in graphic card (Mb) | 113 | 11 |
| Per-cycle runtime (avg. over 20) (min) | 13 | 4 |

**Table S5**. Performance of GRAAL and instaGRAAL at scaffolding the *Ectocarpus* sp. genome.


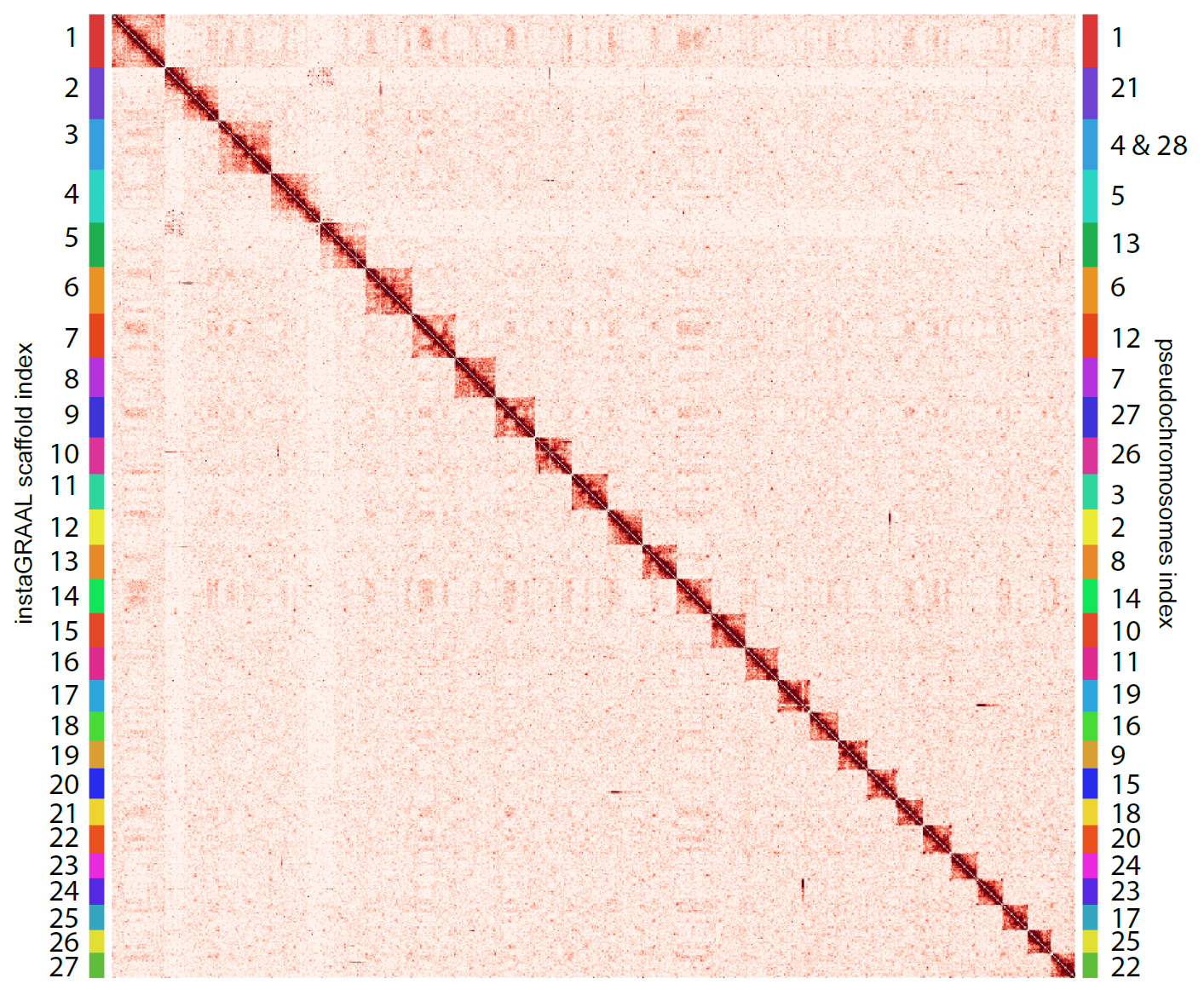


**Figure S1**: Normalized contact map of the *Ectocarpus* sp. genome scaffolded using instaGRAAL (bin = 200 kb). The colour scale represents the normalized interaction frequencies. No large-scale rearrangements are clearly apparent in the interchromosomal contacts. On the right the linkage groups indices from the v2 assembly are indicated.


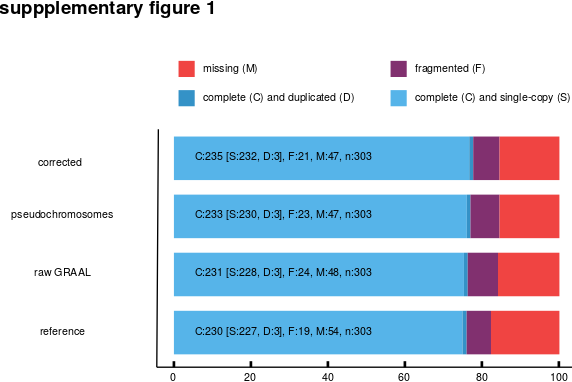


**Figure S2**: estimates of BUSCO-completeness for the three *Ectocarpus* sp. assemblies and the reference genome v1 assembly.


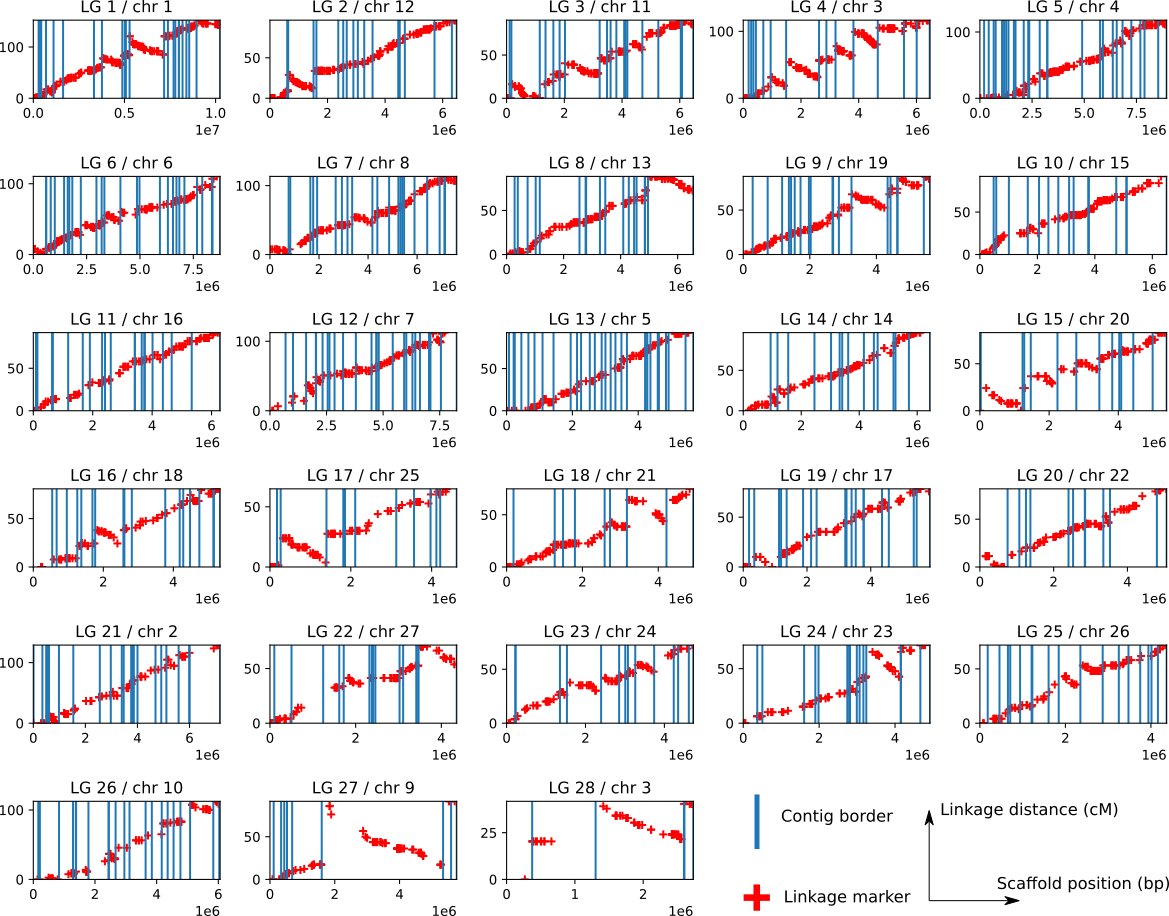


**Figure S3:** Linkage markers *vs.* scaffold positions for all linkage groups/chromosomes (chromosome 3 is made up of linkage groups 4 and 28). The initial contig borders within each chromosome have been underlined. Linkage marker positions are always monotonous (only increasing, or only decreasing) within an initial contig.


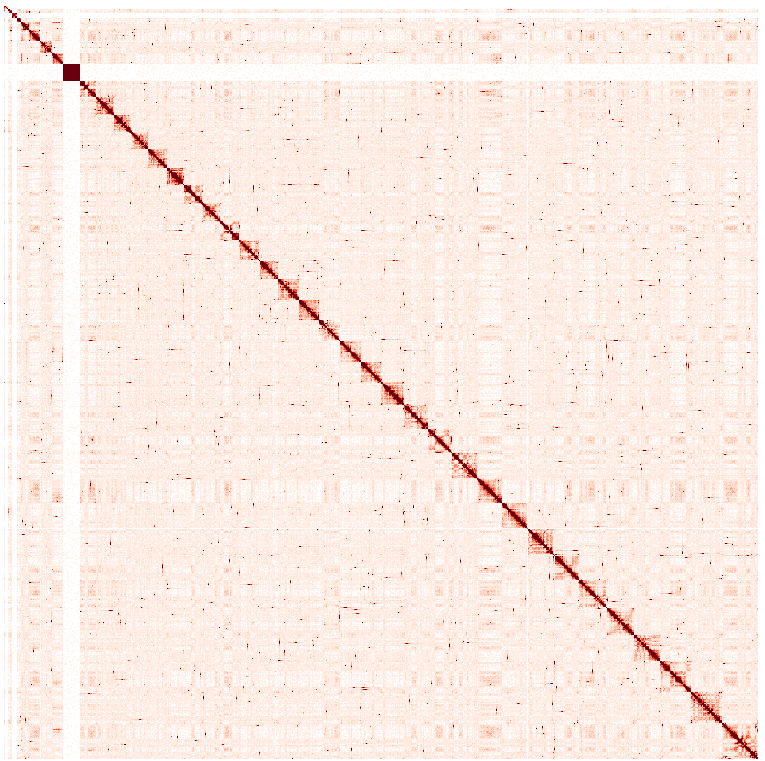
**Figure S4:** The 40 main scaffolds of *Desmarestia herbacea* after instaGRAAL scaffolding.


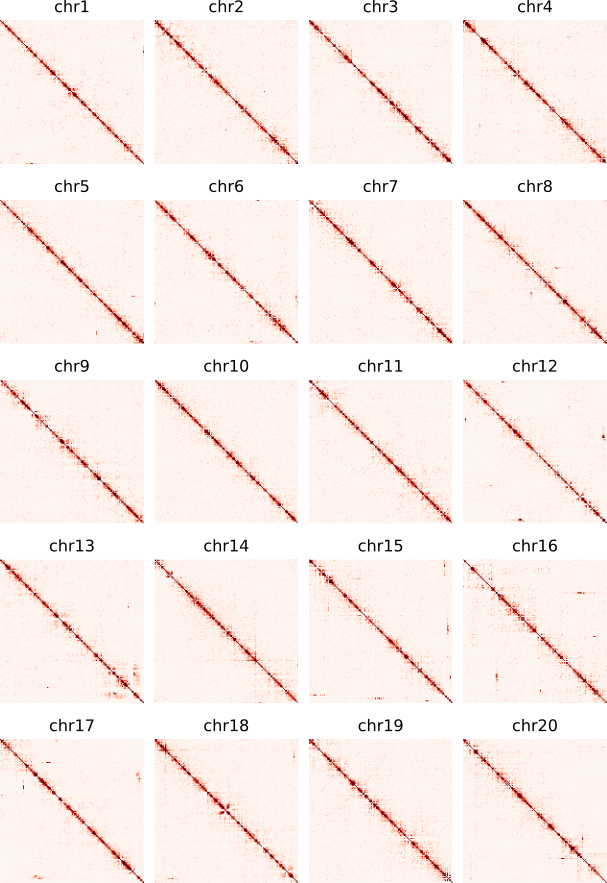


**Figure S5:** Contact maps of the first twenty newly formed scaffolds/putative chromosomes of *Desmarestia herbacea*, generated after scaffolding at a 20 kb resolution.


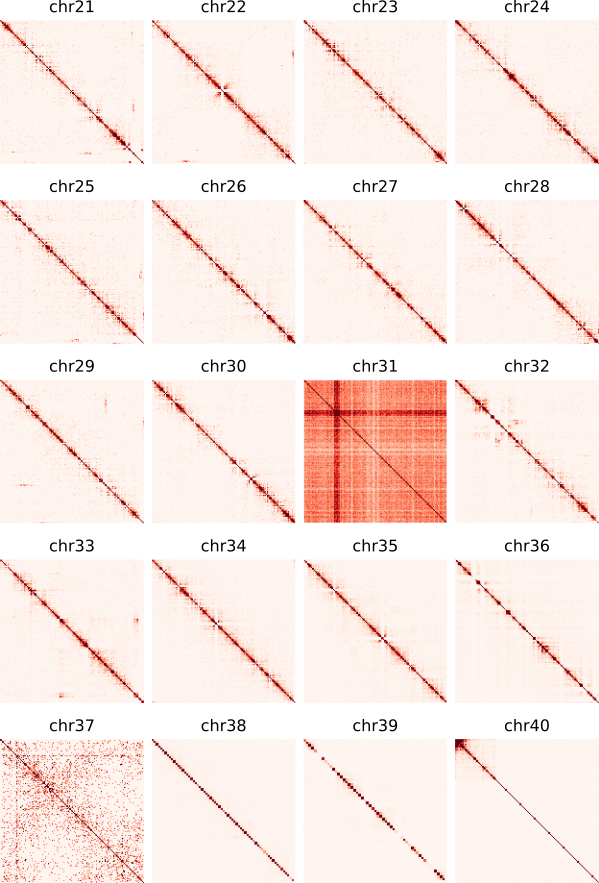


**Figure S6:** The last twenty newly formed scaffolds/putative chromosomes of *Desmarestia herbacea.* post-scaffolding at a 20kb resolution.


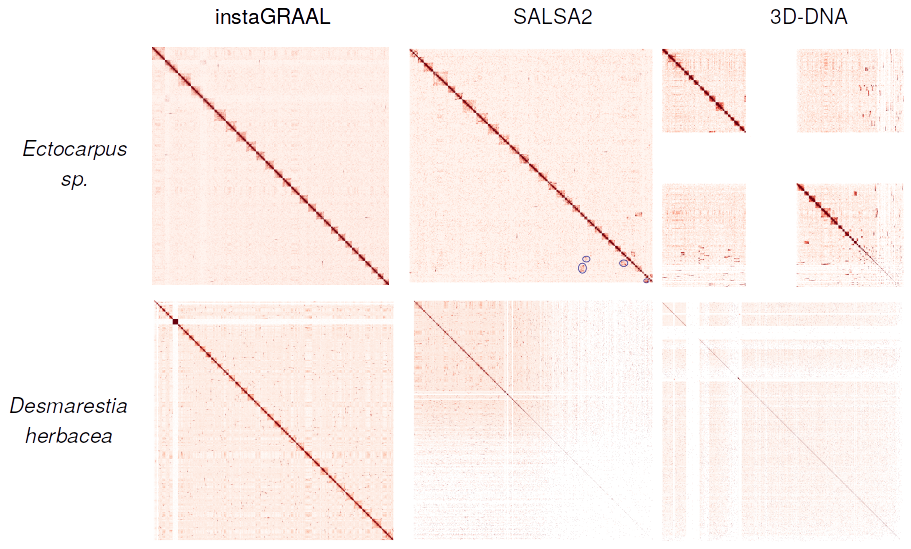
**Figure S7**: Comparisons of contact maps for three different scaffolders across two species. Gaps represent repeated sequences. Scaffolding differences between the SALSA2 vs. instaGRAAL contact maps on *Ectocarpus sp.* are underlined with blue circles in the SALSA2 map.


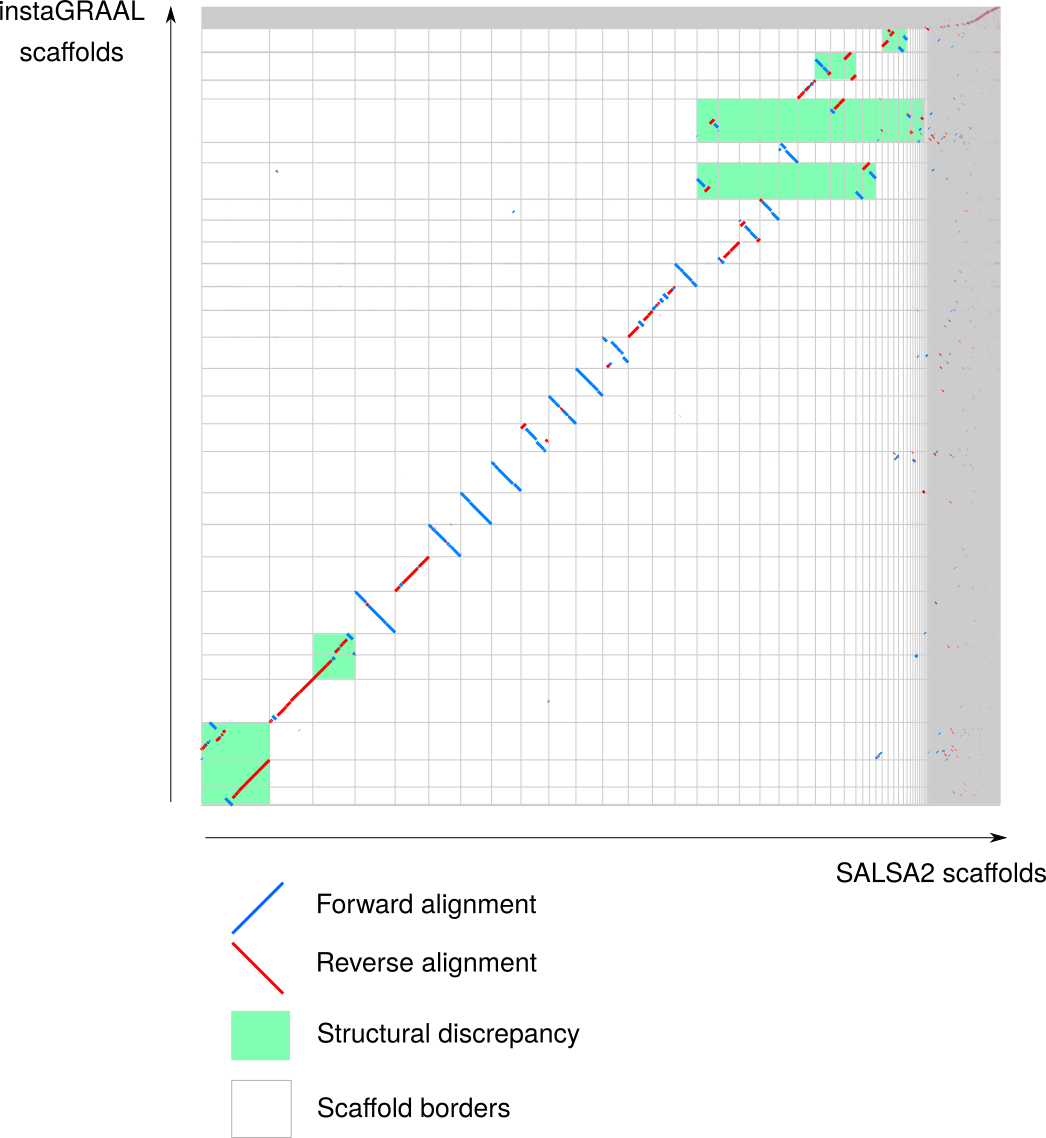
**Figure S8**: Similarity dotplot of the SALSA2 vs. instaGRAAL 27 scaffolds for *Ectocarpus* sp*.*Large-scale structural discrepancies have been underlined in green. The contact maps suggest instaGRAAL solutions are more likely.


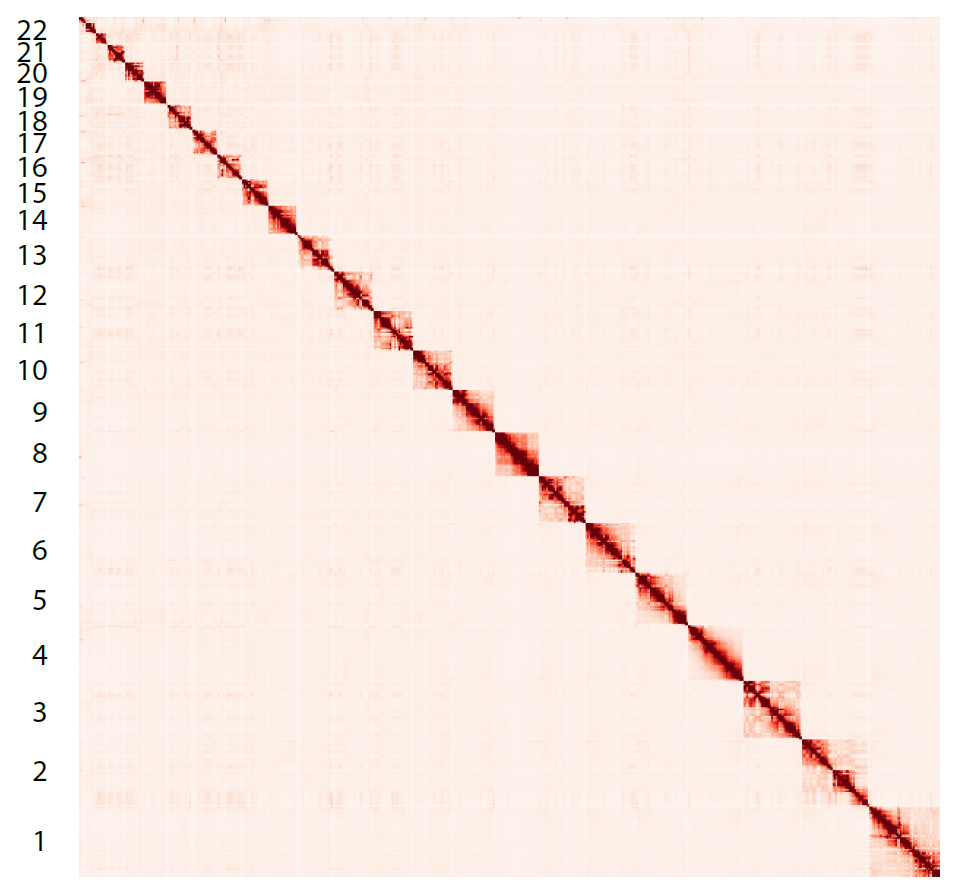
**Figure S9 :** Contact map of the *Homo sapiens* genome, fragmented in 300 kb sequences, after scaffolding with instaGRAAL, at 5-Mb resolution.


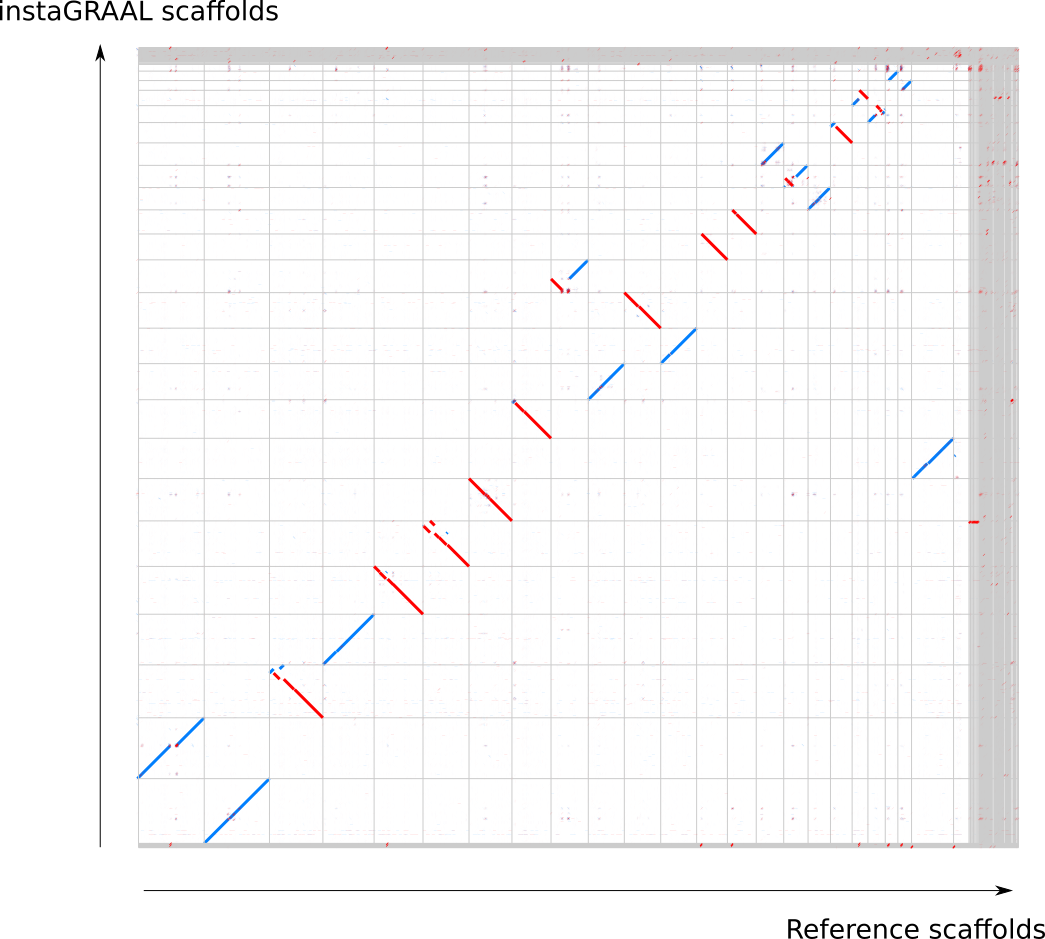


**Figure S10**: Similarity dotplot of the instaGRAAL *vs.* reference scaffolds for the GRCh38 human genome. Relocations are visible but the one-to-one mapping between the 23 first scaffolds is preserved.


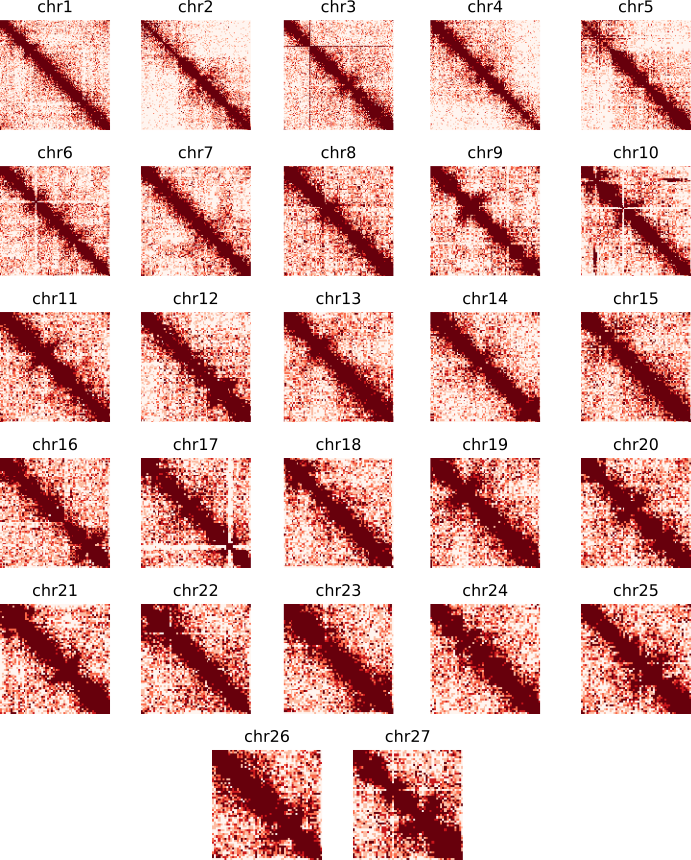


**Figure S11:** All 27 newly formed scaffolds/putative chromosomes of *Ectocarpus sp.* post-scaffolding at a 50kb resolution. Centromere patterns are clearly apparent in all chromosomes, but some errors (potentially due to mapping issues) linger, such as chromosome 10 or 17.
