# Supplementary material for: instaGRAAL: chromosome-level quality scaffolding of genomes using a proximity ligation-based scaffolder
Source: Genome Biol. 2020 Jun 18;21:148. doi: 10.1186/s13059-020-02041-z (PMC7386250; doi:10.1186/s13059-020-02041-z)
Supplement: Supplementary file 2 — Additional file 2. Review history. [file 13059_2020_2041_MOESM2_ESM.docx]

Review History

**First round of review**

**Reviewer 1**

**Were you able to assess all statistics in the manuscript, including the appropriateness of statistical tests used?**

There are no statistics in the manuscript.

**Were you able to directly test the methods?**

No

**Comments to author:**

This paper presents an update to the GRAAL genome scaffolder, called instaGRAAL. InstaGRAAL is one of the few scaffolders that are designed to use Hi-C long range sequencing libraries to produce chromosome scale scaffolds.

Major comments

- One of the main advantages over GRAAL is quoted as being that instaGRAAL can scaffold much larger genomes so will have more widespread utility. I was disappointed to see that the genomes tested in the paper were only a few hundred Mbp in size. If scaling to larger eukaryote genomes is a major advantage for instaGRAAL, I would expect that performance on a human genome (or similar mammalian size genome) would be part of the evaluations performed.

- Most large eukaryote genomes are diploid and the vast majority of new assembly tools now attempt to model the two haplotypes with varying success. The genomes tested in this paper are haploid, therefore the reader cannot infer how this scaffolder might perform on a diploid genomes OR if it has the ability to model or output regions where two haplotypes diverge significantly.

- The performance of instaGRAAL over GRAAL as given in Table 1 is not very convincing that instaGRAAL is a major advancement over GRAAL. Clearly it has had usability and computational resource improvements so that it can run on larger genomes, and has the addition of the polishing step to resolve any resulting misassemblies. I'm not sure these warrant a full publication in Genome Biology, however the editor can decide this.

Minor comments

- Opening paragraph of the background contains terms that are too vague, e.g. 'conventional assemblers', 'pipelines often encounter difficulties'. The language could be tightened a bit more to be more specific. The paragraph feels like it is referring to assemblies from second generation sequencing, whereas many of the new genomes produced now come from third generation long reads with multi megabase contigs. The authors need to be more specific.

- 'Hi-C scaffolds large eukaryotic DNA regions' - I have no idea what this means. What is a 'DNA region'?

- 'genetic linkage data' is used in the evaluation, where is this available from? It is not listed in the Data Availability section of the paper

- the opening sentence of the discussion talks about how instaGRAAL can process 'large eukaryotic genomes' - but the paper does not demonstrate this! The genomes tested are small genomes of a few hundred Mbp!

- the term 'reference contigs' is used in several places. I was unclear as to whether this referred to a reference genome or the input contigs before scaffolding. Please clarify.

**Reviewer 2**

**Were you able to assess all statistics in the manuscript, including the appropriateness of statistical tests used?**

Yes, and I have assessed the statistics in my report.

**Were you able to directly test the methods?**

No

**Comments to author:**

In this manuscript, Baudry et al. present instaGRAAL, a new Hi-C based scaffolding algorithm. Hi-C based scaffolding is currently the most popular approach for establishing super-scaffolds and pseudomolecules in eukaryotic genome assembly projects. Therefore, any tool which demonstrates meaningful advantages over current methods, which can be prone to misassemblies, is of great importance and is worthy of publication.

In making enhancements to their previous software, GRAAL, the authors of instaGRAAL demonstrate a compelling case that they have improved the state-of-the-art in Hi-C based scaffolding. Though improving memory requirements and usability are of much practical importance, their most valuable advantage is in improving the overall structural accuracy of scaffolds. Additionally, the authors show that instaGRAAL can be used on larger genomes, thus inviting a larger portion of the community to utilize their software. The discussion section will also be valuable for helping potential users make important decisions about the Hi-C process, especially how the choice of restriction enzyme can affect scaffolding. Finally, the ability to use linkage data to improve scaffolding is a very nice feature.

The work is overall high quality, although there are a few critical aspects that should be addressed prior to publication and that I have outlined below.

Major Points:

- In the manuscript, the authors assemble 2 haploid genomes in order to evaluate and assess instaGRAAL. However, the use of haploid genomes ignores a major complexity that most researchers face in eukaryotic genome assembly projects. Demonstrating positive scaffolding results for a well established diploid genome would present a much more convincing case for the efficacy of this tool.

- Quast is used to evaluate misassemblies in the Ectocarpus assemblies, though it is unclear how it was used. What was the reference genome used with Quast? Does such a reference exist for Ectocarpus? Or was Quast used without a reference? In short, the manuscript could use some details describing to the reader why Quast is a useful tool to measure misassemblies in this case?

- A novel aspect of the software is that it ultimately scaffolds bins of restriction fragments rather than contigs. In this mode, how many contigs are reconstructed exactly by instaGRAAL? In other words, if a contig is initially split up into discrete bins, how often is the original intact contig reconstructed in the final pseudomolecules? This would be a good measurement for scaffolding success. Or, it can be used as evidence for contig-level misassembly correction if a contig remains broken, and that break is ultimately supported by the Hi-C data.

- To convince the community of the absolute and relative structural accuracy of assemblies, more visual aids are required. Firstly, as you have included in figures S2, S3 and S4, Hi-C heatmaps of the assemblies should be presented. However, these supplementary figures are too far zoomed-out to be able to see potential misassemblies within scaffolds. Such plots for individual scaffolds would help readers see the details of the assembly structure. Relatedly, it would be very valuable to plot the linkage maps against the scaffolded assemblies for the Ectocarpus assemblies to demonstrate overall structural accuracy. Specifically, this plot would align the linkage-map markers to the scaffolds, and plot the linkage map position (cM, y-axis) against the scaffold position (bp, x-axis).

Minor Points

- The caption for Figure 1, subfigure b.iv is missing

- instalGRAAL can process larger genomes than GRAAL, but how much larger? Can it be used to scaffold a human genome assembly? It would be useful if readers could know before hand if their assemblies qualify.

- Memory and runtime comparisons between GRAAL and instaGRAAL should be recorded in a supplementary table for the Ectocarpus assemblies since these are supposed to be major improvements in the software.

- The authors should avoid the term "polishing" when describing the new feature of instaGRAAL since "polishing" is associated with improving base-level consensus accuracy of genome assemblies (e.g. Racon and Pilon). This feature would be better described as "misassembly correction".

- The authors comment: "Therefore, our benchmarking attempt should be rather seen as a way to stress the importance of implementing sensible default parameters that readily cover as many use cases as possible for the end user." However, this could also be seen as potentially overfitting for these datasets, especially since one 2 genomes are presented. The authors should provide more details for the data requirements for high quality scaffolding.

- The paper seems to be presenting these genome assemblies as references to the community. If so, the assemblies should be held up to usual scrutiny, e.g. polishing and bacterial contamination screening should be performed. Also, the assemblies should be made publicly available.

**Authors Response**

**Point-by-point responses to the reviewers’ comments:**

*We are pleased to submit a revised version of our manuscript “Chromosome-level quality scaffolding of brown algal genomes using InstaGRAAL, a proximity ligation-based scaffolder”, which describes instaGRAAL, a program that exploits chromosome conformation capture (Hi-C) data to scaffold genomes of any size, including large genomes. We have revised the work according to the reviewers' requests, and we think that the manuscript is now substantially improved.*

*In order to respond to the referees' questions and suggestions:*

*We have further validated instaGRAAL using the human genome as a test case. We show that InstaGRAAL can scaffold with high fidelity the human genome into 22 autosomes following fragmentation into 300 kb pieces (plus the X chromosome).*

*We improved the assessment of the time and the computer resources needed to run instaGRAAL, to provide the reader with an idea of the running costs of the program.*

*We discuss more thoroughly the effects of diploidy or polyploidy on the results of the program. We show, in the response to referee 1, that instaGRAAL is able to process haplotypes of a allopolyploid genome. This demonstration, which supports our discussion, cannot be included in the present manuscript as this genome is currently being analysed for an independent publication.*

*We benchmarked instaGRAAL against other available solutions. This benchmarking will help the user to understand the limitations and caveats of the final assembly, avoiding the misled belief that Hi-C scaffolding is a miraculous solution that is out of reach for the common laboratory.*

*Finally, we have corrected and improved the manuscript throughout according to the referees’ suggestions. We hope you agree that the resulting manuscript is much improved, compared to the original submission.*

*Overall, given the multiple projects ongoing in the genomic field, we strongly believe that robust, open-source programs must be made available to the broad community of interested scientists: instaGRAAL responds to this need.*

*The results were achieved by simply running the program with default parameters on a laptop computer.*

*Below is the point by point response to the reviewer’s comments.*

Reviewer reports:

Reviewer #1: This paper presents an update to the GRAAL genome scaffolder, called instaGRAAL. InstaGRAAL is one of the few scaffolders that are designed to use Hi-C long range sequencing libraries to produce chromosome scale scaffolds.

Major comments:

One of the main advantages over GRAAL is quoted as being that instaGRAAL can scaffold much larger genomes so will have more widespread utility. I was disappointed to see that the genomes tested in the paper were only a few hundred Mbp in size. If scaling to larger eukaryote genomes is a major advantage for instaGRAAL, I would expect that performance on a human genome (or similar mammalian size genome) would be part of the evaluations performed.

*We acknowledge that the genomes we assembled in the previous version of the manuscript, while relatively large and still an order of magnitude higher than those assembled using GRAAL, were still sub-Gb sized. We have, therefore, addressed this concern by scaffolding a human genome and verifying that the newly formed scaffolds match the twenty-three chromosomes. These results are described in Table 2, Figure S10, Figure S11 of the revised version.*

Most large eukaryote genomes are diploid and the vast majority of new assembly tools now attempt to model the two haplotypes with varying success. The genomes tested in this paper are haploid, therefore the reader cannot infer how this scaffolder might perform on a diploid genomes OR if it has the ability to model or output regions where two haplotypes diverge significantly.

*This is an excellent point. So far, haplotyping remains a challenge in the field. Logically, the capacity of instaGRAAL to successfully scaffold a diploid genome is expected to be dependent on the quality of the starting assembly. If the matching and diverging regions of a diploid starting assembly are contained within the same scaffold (or better yet, within a single contig), the genome will be efficiently assembled and phased by instaGRAAL provided that there is sufficient binning to allow the signatures of each haplotype (i.e. the corresponding contact map block of the matching + diverging region for each haplotype) to be distinguished. On the other hand, if the matching and diverging regions are located in different scaffolds, there will not be sufficient prior information to create a phased assembly. We have confirmed these predictions using another genome that was not part of our present study and is the focus of an upcoming publication.*

*For the purpose of this revision, we confidentially show below (Figure 1) the results of instaGRAAL scaffolding of the [redacted]. The original assembly with Mira [redacted] resulted in a number of hybrid, misassembled contigs. InstaGRAAL was able to cluster these contigs into chromosomes, but could not phase haplotypes as they were intermingled (Figure 1, left panel). A new assembly generated using BWISE produced contigs that were subsequently scaffolded by instaGRAAL into a complete, phase genome assembly (Figure 1, right panel). We discuss this point in the instaGRAAL manuscript, without showing the unpublished results which are part of another, exhaustive study.*

*Figure 1. Comparison between the instaGRAAL outputs obtained de novo with an unsegregated MIRA assembly (left panel) and a segregated BWISE assembly (right panel; https://github.com/Malefoy/Bwise). Bwise is an assembler specifically designed for de novo polyploid assembly and instaGRAAL preserves the haplotype information. The outputs were obtained using the same instaGRAAL parameters. Top panels: contact maps of the largest scaffolds obtained after scaffolding. Bottom panel: dot-plot comparisons of scaffolds. The numbers on the Y axis indicate pairs of homologs in the maps. In the MIRA assembly, intermingled hybrid contigs resulted in unique chromosomes. In the Bwise assembly, the quality of the contigs was sufficient to phase the two different haplotypes into full-length scaffolds for five out of six pairs of homologs (chromosomes). The last pair of homologs (6+6’) are highly similar in this species and could not be phased.*

*Based on this analysis, we would argue that phasing capacity depends critically on the quality of the starting assembly. A related point was stressed in the discussion of the revised manuscript, where we underline the importance of not having highly fragmented starting assemblies because the size distribution distorts the contact distribution. In other words, instaGRAAL preserves haplotype information and successfully produces phased chromosomes if two sets of roughly matching scaffolds are given, but cannot determine haplotypes if the starting data is not of sufficient quality (“garbage in, garbage out”).*

The performance of instaGRAAL over GRAAL as given in Table 1 is not very convincing that instaGRAAL is a major advancement over GRAAL. Clearly it has had usability and computational resource improvements so that it can run on larger genomes, and has the addition of the polishing step to resolve any resulting misassemblies. I'm not sure these warrant a full publication in Genome Biology, however the editor can decide this.

*GRAAL, developed more than 7 years ago, was a proof of concept with usability issues. Notably, it could not be applied to large genomes because of its need in computational resources. That made it impractical for larger use by the community. The instaGRAAL version is readily usable and, in fact, has already been integrated into various assembly projects of large genomes. Given the small number of usable programs developed and available in this field, we think it is important to offer the community a broader range of options regarding this important step of genomic projects.*

Minor comments

Opening paragraph of the background contains terms that are too vague, e.g. 'conventional assemblers', 'pipelines often encounter difficulties'. The language could be tightened a bit more to be more specific. The paragraph feels like it is referring to assemblies from second generation sequencing, whereas many of the new genomes produced now come from third generation long reads with multi megabase contigs.

*Both of the genomes we scaffolded were generated with reads from second generation sequencing, and our choice of words may have reflected this. However, we have successfully used instaGRAAL on genomes generated with third generation long reads, and almost all of our reasoning is independent of the generation of technology used. As a Hi-C scaffolder, instaGRAAL does not depend on the method used to generate the starting assembly. We have modified the introduction to take this comment into account.*

·	'Hi-C scaffolds large eukaryotic DNA regions' - I have no idea what this means. What is a 'DNA region'?

*By ‘DNA region’ we mean any size stretch of base pairs. We used this general terminology to designate any such stretch, from groups of restriction fragments to contigs to entire scaffolds. We now explicitly define the term.*

·	'genetic linkage data' is used in the evaluation, where is this available from? It is not listed in the Data Availability section of the paper

*This was an oversight on our part and we apologize for it. We have now uploaded this data to https://github.com/koszullab/ectocarpus_scripts, which serves as a general purpose repository to reproduce the main results of our papers.*

·	the opening sentence of the discussion talks about how instaGRAAL can process 'large eukaryotic genomes' - but the paper does not demonstrate this! The genomes tested are small genomes of a few hundred Mbp!

*As noted above, we have addressed this point and the revised manuscript presents successful scaffolding of a human genome.*

·	the term 'reference contigs' is used in several places. I was unclear as to whether this referred to a reference genome or the input contigs before scaffolding. Please clarify.

*We agree that the terms we use are ambiguous. We are now careful to use “input contigs” to refer to contigs resulting from the original assembly step, whether they are subsequently scaffolded or not. We have went through the manuscript to remove ambiguities.*

Reviewer #2: In this manuscript, Baudry et al. present instaGRAAL, a new Hi-C based scaffolding algorithm. Hi-C based scaffolding is currently the most popular approach for establishing super-scaffolds and pseudomolecules in eukaryotic genome assembly projects. Therefore, any tool which demonstrates meaningful advantages over current methods, which can be prone to misassemblies, is of great importance and is worthy of publication.

In making enhancements to their previous software, GRAAL, the authors of instaGRAAL demonstrate a compelling case that they have improved the state-of-the-art in Hi-C based scaffolding. Though improving memory requirements and usability are of much practical importance, their most valuable advantage is in improving the overall structural accuracy of scaffolds. Additionally, the authors show that instaGRAAL can be used on larger genomes, thus inviting a larger portion of the community to utilize their software. The discussion section will also be valuable for helping potential users make important decisions about the Hi-C process, especially how the choice of restriction enzyme can affect scaffolding.

Finally, the ability to use linkage data to improve scaffolding is a very nice feature. The work is overall high quality, although there are a few critical aspects that should be addressed prior to publication and that I have outlined below.

In the manuscript, the authors assemble 2 haploid genomes in order to evaluate and assess instaGRAAL. However, the use of haploid genomes ignores a major complexity that most researchers face in eukaryotic genome assembly projects. Demonstrating positive scaffolding results for a well-established diploid genome would present a much more convincing case for the efficacy of this tool.

*We acknowledge that diploid genomes are important to consider. In the revised manuscript, we discuss the point that the quality of the scaffolding of a diploid genome will depend on the degree of divergence between the two haplotypes and the quality of the original assembly. This point and further validation is discussed in detail in our reply to reviewer 1.*

Quast is used to evaluate misassemblies in the Ectocarpus assemblies, though it is unclear how it was used. What was the reference genome used with Quast? Does such a reference exist for Ectocarpus? Or was Quast used without a reference? In short, the manuscript could use some details describing to the reader why Quast is a useful tool to measure misassemblies in this case?

*We apologize for the lack of clarity. There were also ambiguities in our use of “reference” throughout the text, which we have now alleviated (see below as well).*

*Quast was indeed used with a reference genome of Ectocarpus (Courmier et al., 2017). We used this scaffold as the reference, original assembly that was used for the scaffolding by instaGRAAL. We chose to use Quast because it is an evaluation tool known for its comprehensiveness, taking as input a large number of diverse data sources and outputting a broad range of the relevant metrics, and it is largely used by the broader community. We acknowledge that the misassembly screening it uses is relatively superficial and does not carry out fine-grained high-resolution error checking, but we believe that this type of analysis outside the scope of a paper focused on Hi-C scaffolding, and as long as improvements are observed with the same metric as measured by the same software, the result should still be noteworthy. Corrected and polished assemblies beyond instaGRAAL’s use of Hi-C or linkage disequilibrium should be carried out using specific software such as Racon or HyPo. We now discuss this point in the text.*

A novel aspect of the software is that it ultimately scaffolds bins of restriction fragments rather than contigs. In this mode, how many contigs are reconstructed exactly by instaGRAAL? In other words, if a contig is initially split up into discrete bins, how often is the original intact contig reconstructed in the final pseudomolecules? This would be a good measurement for scaffolding success. Or, it can be used as evidence for contig-level misassembly correction if a contig remains broken, and that break is ultimately supported by the Hi-C data.

*This is a very interesting question and indeed a hard problem. Our scaffolder’s strength is indeed that it can break up contigs into bins or fragments, or choose to keep the existing structure in fine post-scaffolding. Other scaffolders, by comparisons, do not modify the initial contig structure at all. This choice is understandable, but it means that these scaffolders ignore contig-level misassemblies. On the other hand, the original GRAAL program had a problem in that it broke up contigs too often, and the end result was a set of scaffolds with many local mistakes including flips, inversions and other such small-scale errors. In practice, the initial contig structure is usually trusted, but we also want the ability to ignore it temporarily in order to explore genome space in a way that lets us converge on a close-to-optimal solution. The answer is instaGRAAL, which breaks scaffolds down in order to reassemble a Hi-C-optimal genome, but then reinjects information about the contigs as needed in order to correct any errors introduced during assembly. The process has to be in two phases, so that the initial structure does not bias the Hi-C scaffolder but is still taken into account at the end. Moreover, the “correction” is not binary; some, or all of the putative artefacts can be “corrected” depending on how trustworthy the initial contigs are. A broken down contig can be totally reconstructed, or left as is. Since the strategy employed will depend on the user’s knowledge of the quality of the initial assembly, we leave the decision up to the user. The command-line interface has many of options that can be tested and this process can also be easily integrated in a developer’s workflow as a library. The command options are listed in the manuscript and online.*

*We also want to stress that, generally speaking, we cannot provide a clean breakdown of misassembly detection/correction, due to the very nature of the Hi-C protocol. There are always going to be false junctions since the fundamental unit of our scaffolding is the restriction fragment. Therefore, there is a lower bound on the resolution of scaffolding corresponding to about 1kb (in order of magnitude). As noted above, further fine-grained polishing could be carried out with softwares such as Racon or Pilon.*

- To convince the community of the absolute and relative structural accuracy of assemblies, more visual aids are required. Firstly, as you have included in figures S2, S3 and S4, Hi-C heatmaps of the assemblies should be presented. However, these supplementary figures are too far zoomed-out to be able to see potential misassemblies within scaffolds. Such plots for individual scaffolds would help readers see the details of the assembly structure. Relatedly, it would be very valuable to plot the linkage maps against the scaffolded assemblies for the Ectocarpus assemblies to demonstrate overall structural accuracy. Specifically, this plot would align the linkage-map markers to the scaffolds, and plot the linkage map position (cM, y-axis) against the scaffold position (bp, x-axis).

*We acknowledge these points and have added high-resolution contact maps as requested, as well as a plot of positions of markers vs. scaffold borders.*

Minor Points

- The caption for Figure 1, subfigure b.iv is missing

*We have added the caption to Figure 1 that was missing.*

- instaGRAAL can process larger genomes than GRAAL, but how much larger? Can it be used to scaffold a human genome assembly? It would be useful if readers could know before hand if their assemblies qualify.

*As noted above, we have included a human genome assembly as a benchmark to confirm instaGRAAL can indeed process genomes similarly sized. We have also scaffolded a number of genomes of similar sizes, or lower, without encountering specific issues. The main limitation would therefore consist in the computational power dedicated to the scaffolding (memory, GPU, etc.)*

- Memory and runtime comparisons between GRAAL and instaGRAAL should be recorded in a supplementary table for the Ectocarpus assemblies since these are supposed to be major improvements in the software.

*We have added metrics for per-cycle runtime and total memory load for Ectocarpus sp., which has a reasonably sized genome (Table S5). Note, however, that one of the reasons such metrics were missing was that the initial GRAAL program could not run at all for larger assemblies, mostly due to memory requirements beyond those of a typical desktop computer.*

- The authors should avoid the term "polishing" when describing the new feature of instaGRAAL since "polishing" is associated with improving base-level consensus accuracy of genome assemblies (e.g. Racon and Pilon). This feature would be better described as "misassembly correction".

*We have rectified the wording as noted by the reviewer to be less confusing.*

- The authors comment: "Therefore, our benchmarking attempt should be rather seen as a way to stress the importance of implementing sensible default parameters that readily cover as many use cases as possible for the end user." However, this could also be seen as potentially overfitting for these datasets, especially since one 2 genomes are presented. The authors should provide more details for the data requirements for high quality scaffolding.

*Our comment reflected the fact that we have run instaGRAAL on many different genomes (40+). Extensive trial and error on all these genomes was performed in order to define the default parameters that were the most likely to fit the whole set of test assemblies of various sizes. For various reasons, mostly related to data ownership and ongoing publications, we cannot provide these assemblies in the present paper. In an effort to address this omission, we have added more information about the parameters and data requirements for scaffolding as requested.*

- The paper seems to be presenting these genome assemblies as references to the community. If so, the assemblies should be held up to usual scrutiny, e.g. polishing and bacterial contamination screening should be performed. Also, the assemblies should be made publicly available.

*This is an oversight of our part and has been corrected. Assemblies have been uploaded on https://github.com/koszullab/ectocarpus_scripts, which serves as a general purpose repository to reproduce the main results of our papers. The final scaffolding will be deposited at NCBI as well. The datasets generated and analyzed in the present work are available in the SRA repository, SRR8550777. For Ectocarpus, the v3 will be put onto Orcae and submitted to EBI with the aim of then transferring to Ensembl.*

**Second round of review**

**Reviewer 2**

In their updates to the initial manuscript, the authors substantially improved the demonstration of the utility of instaGRAAL by scaffolding a human genome assembly. They also incorporate some of our suggestions around language. For example, the term “polishing” is no longer used to describe the assembly refinement process.

However, the Ectocarpus scaffolding results are still unclear and/or incomplete. These results, along with several minor points throughout the paper, should be addressed prior to publication. Importantly, the quality of the writing, I believe, is insufficient for publication as it currently stands.

Major Points

The results for the Ectocarpus assembly scaffolding are still unclear. First, I will state what I understand the scenario to be:

In 2010, Cock et al. published the first draft (not a reference) assembly of Ectocarpus siliculosus. For simplicity, let’s call this “ESD”. This assembly was fragmented and had a limited set of gene annotations. Then, in 2017, Cormier et al. ordered and oriented the ESD assembly (via a linkage map) into pseudomolecules to establish the first proper reference genome for Ectocarpus. Let’s call this assembly “ESR”. ESR also had a much-improved gene annotation set. Finally, in your manuscript, you scaffold the ESD assembly with both GRAAL and instaGRAAL. Let’s call the GRAAL scaffolds “ESG” and the instaGRAAL scaffolds “ESI”. Assuming that this summary is correct, I have the following comments about how these results could be more clear, informative and impactful.

- The introduction should outline these assemblies just as if have above. Personally, I don’t think it is proper to call ESD a “reference” since it was only ever a fragmented draft assembly with limited annotations. Rather, ESR is the “reference” genome for Ectocarpus.

- The manuscript states that “The Ectocarpus genome is currently only published in draft form”. But that is not true since the ESR assembly, published in 2017, is a proper reference assembly. I would argue that ESD is a draft assembly and ESR is a reference assembly.

- “Scaffolding of the Ectocarpus sp. chromosomes with instaGRAAL” and “Convergence of the Ectocarpus sp. assembly towards 27 major scaffolds” should be combined into one section.

- The section “Correcting the chromosome-level instaGRAAL assembly of the Ectocarpus sp. Genome” requires some additional attention. Sometimes ESD is referred to as “the original assembly”, which is vague. I recommend assigning labels to the assemblies as I have done.

- Also in this section, it is unclear how the user invokes the assembly correction/refinement process. Is it automatic, or a separate module? And what exactly is it called now? In my initial review, I suggested against using the word “polish”, but now I don’t see what word has replaced it. Perhaps you are using the word “refinement”? This should all be emphasized in the introduction since it is a major feature improvement to GRAAL.

- Additionally for this section, the manuscript states “To further validate the assembly, we exploited genetic linkage data to search for potential translocations between scaffold extremities [26]. This analysis, now implemented as an option in instaGRAAL, detected such events in the uncorrected version but none in the corrected assembly”. But there is no data to support this, such as a table or a figure. How can the reader know that the linkage map did not detect any misassemblies in the corrected assembly?

- I believe the section entitled “Comparisons with previous Ectocarpus sp. assemblies and validation of the instaGRAAL assembly” is misguided, and I believe the entire analysis needs to be reconsidered. I have 3 primary suggestions:

ESR should be used as the “reference” assembly. This is because ESR has a much more complete set of gene annotations. Therefore, one could try to map these annotations onto ESG or ESI as a measurement of accuracy. I don’t believe it is valid to map the less complete annotations from ESD, as is done in the current manuscript.

ESD should not be included in this analysis. A sufficient comparison to ESD and ESI is already covered in “Correcting the chromosome-level instaGRAAL assembly of the Ectocarpus sp. Genome”. If you want to include a ESD/ESG comparison, include it in that section.

QUAST should not be used here. QUAST requires a reference genome, and if you take my advice from the earlier, the reference would be ESR. But the whole point of this analysis is that ESR may be less structurally accurate than ESG or ESI. In genomic regions that don’t agree with each other in these assemblies, one does not know which assembly is correct, and therefore using ESR as a reference in QUAST is invalid. Rather, as was done in the previous sections, one should use the linkage map as an independent validation set. For example, one can align ESR, ESG and ESI to the linkage map and see which assembly shows the most concordance. You currently make use of the linkage map in this section, but just as before, there is no figure or table to show these results. As another potential independent validation, one could also align the Hi-C data to ESR/ESG and compare the contact frequency maps.

- “Comparison between the Ectocarpus sp. instaGRAAL and pseudochromosomal assemblies” should be incorporated into the end of “Convergence of the Ectocarpus sp. assembly towards 27 major scaffolds”. I am not sure it warrants its own section.

- QUAST should be used to assess the human genome scaffolding success. In this scenario, the human reference would be used as the “reference” and the instaGRAAL scaffolds would be used as the “query”. This would provide a nice quantitative assessment to supplement the qualitative assessment provided in the manuscript.

- A reference to Table S4 should be placed after the following sentence: “ Sequencing reads were processed using the hybrid MaSuRCA assembler (v3.2.9) [31], yielding 7,743 contigs representing 496 Mb”. Furthermore, 15X coverage of long reads is insufficient and may explain the poor assembly quality. In the discussion, the authors state “This is only a problem if one chooses not to reconstruct every input contig within a newly formed scaffold with our correction procedure, i.e. one is distrustful of the initial input contigs. This was not the case for Ectocarpus sp. but could be argued for D. herbacea, where the de novo contigs generated from an hybrid assembly are more error-prone”. But there is no evidence that MaSuRCA assemblies are more “error-prone” because they are hybrid assemblies, and I have never seen such claims in the literature. Rather, high-quality MaSuRCA assemblies are regularly published.

- The section entitled “Integrating information from the Hi-C analysis with other types of data” seems unnecessary and out of scope for this particular manuscript.

- The section entitled “Validation with the genetic map” is unclear. Firstly, I believe the word “pseudochromosome” is incorrectly used in place of “linkage group”. The authors state “It then finds best-matching pairs of pseudochromosomes/scaffolds by counting how many of these regions overlap from one set to the other”, but what is meant by “overlap”. How do Hi-C links overlap with marker pairs?

Minor Points

- The manuscript states “... we ran SALSA2 [33] and 3D-DNA on the same Ectocarpus sp. and Desmarestia herbacea reference genome and Hi-C reads”. Again this is a misuse of the word “reference”. I believe SALSA2 and 3D-DNA scaffolded the ESD genome, which is not a reference, along with the Desmarestia genome presented in this paper, which is also not a reference genome.

- The manuscript states “InstaGRAAL was also more efficient than the genetic map in orienting scaffolds (Table S2)”. But I believe “efficient” should be replaced with “accurate”.

- The discussion section “Reference genome assembly based correction” is misleading. I believe this refers to instaGRAAL’s “refinement” step and has nothing to do with a reference genome.

- In general, the writing throughout the manuscript needs to be improved, specifically in terms of grammar and organization. Here are some examples:

“The Hi-C scaffolder GRAAL (Genome Re-Assembly Assessing Likelihood from 3D) is a probabilistic tool that uses a Markov Chain Monte Carlo (MCMC) approach was able to generate the first chromosome-level assembly of an incomplete eukaryotic genome [13] by permuting DNA segments according to their contact frequencies until the most likely scaffold was reached (see also [15]).” - This sentence starts off in the present tense and ends in the past tense. It also looks like it is missing a few words between “approach” and “was able”.

The word “exploit” is over-used.

“The remainder of the cycles are devoted to intra-chromosomal refinement”. - Here, “are” should be “is”.

“99.4% of the sequences in the 1,018 contigs was integrated into the 27 largest scaffolds after instaGRAAL processing.” - Here, “was” should be “were”.

.

**Authors Response**

**Point-by-point responses to the reviewers’ comments:**

Point by point response to reviewer’s comments

Reviewer #2: In their updates to the initial manuscript, the authors substantially improved the demonstration of the utility of instaGRAAL by scaffolding a human genome assembly. They also incorporate some of our suggestions around language. For example, the term “polishing” is no longer used to describe the assembly refinement process.

However, the Ectocarpus scaffolding results are still unclear and/or incomplete. These results, along with several minor points throughout the paper, should be addressed prior to publication. Importantly, the quality of the writing, I believe, is insufficient for publication as it currently stands.

Major Points

The results for the Ectocarpus assembly scaffolding are still unclear. First, I will state what I understand the scenario to be:

In 2010, Cock et al. published the first draft (not a reference) assembly of Ectocarpussiliculosus. For simplicity, let’s call this “ESD”. This assembly was fragmented and had a limited set of gene annotations. Then, in 2017, Cormier et al. ordered and oriented the ESD assembly (via a linkage map) into pseudomolecules to establish the first proper reference genome for Ectocarpus. Let’s call this assembly “ESR”. ESR also had a much-improved gene annotation set. Finally, in your manuscript, you scaffold the ESD assembly with both GRAAL and instaGRAAL. Let’s call the GRAAL scaffolds “ESG” and the instaGRAAL scaffolds “ESI”. Assuming that this summary is correct, I have the following comments about how these results could be more clear, informative and impactful.

*RE: We propose to call these different assemblies v1, v2, v3,v4 for simplicity, and have modified the manuscript accordingly.*

*V1 = assembly using sanger and genetic map (Cock et al 2010 + Heesch et al 2010)*

*V2= amelioration of V1 by using RANseq data ans RADseq genetic map (Cormier et al. 2016)*

*V3= instaGraal assembly*

*V4= instaGraal assembly + correction*

*We disagree that the v1 (Cock et al.) is a ‘draft’, as it contains already a large number of genes manually annotated (and some of us would strongly disagree with the argument, given they contributed to the work at the time). Moreover, the v1 was also based on a genetic map (Heesch et al; 2010). Overall, we would like to maintain the reference to this version of the genome as the v1 assembly.*

*V2 was indeed an improved version of the genome because in the meantime a large amount of RNAseq data were generated that substantially improved the annotation. The genetic map was also improved, allowing to anchor more contigs than in the v1 (with more markers, this was a Rad-seq map). But overall this is expected, as years pass genome assemblies are being refined and improved. Only recently have nearly full genomes been obtained in their first sequencing efforts, thanks to technological advances.*

*Therefore, we would like to maintain the designation of the first genome from Cock et al.,2010 as the v1 assembly of the Ectocarpus genome.*

- The introduction should outline these assemblies just as if have above. Personally, I don’t think it is proper to call ESD a “reference” since it was only ever a fragmented draft assembly with limited annotations. Rather, ESR is the “reference” genome for Ectocarpus.

*RE: We have outlined the assemblies as requested in the introduction. Major metrics are already found in Table 2.*

- The manuscript states that “The Ectocarpus genome is currently only published in draft form”. But that is not true since the ESR assembly, published in 2017, is a proper reference assembly. I would argue that ESD is a draft assembly and ESR is a reference assembly.

*RE: The referee is right! We modified the sentence accordingly.*

- “Scaffolding of the Ectocarpus sp. chromosomes with instaGRAAL” and “Convergence of the Ectocarpus sp. assembly towards 27 major scaffolds” should be combined into one section.

*RE: We implemented the change.*

- The section “Correcting the chromosome-level instaGRAAL assembly of the Ectocarpus sp. Genome” requires some additional attention. Sometimes ESD is referred to as “the original assembly”, which is vague. I recommend assigning labels to the assemblies as I have done.

*RE: We now systematically call the original assembly “v1 assembly”.*

- Also in this section, it is unclear how the user invokes the assembly correction/refinement process. Is it automatic, or a separate module? And what exactly is it called now? In my initial review, I suggested against using the word “polish”, but now I don’t see what word has replaced it. Perhaps you are using the word “refinement”? This should all be emphasized in the introduction since it is a major feature improvement to GRAAL.

*RE: We now have clarified the implementation of this correction step. The word polished was replaced by “correction”. We implemented it as a separate “correction” module that is automatically installed alongside the scaffolder. Detailed implementation specifics are to be found on the github repository, and in instaGRAAL’s documentation.*

- Additionally for this section, the manuscript states “To further validate the assembly, we exploited genetic linkage data to search for potential translocations between scaffold extremities [26]. This analysis, now implemented as an option in instaGRAAL, detected such events in the uncorrected version but none in the corrected assembly”. But there is no data to support this, such as a table or a figure. How can the reader know that the linkage map did not detect any misassemblies in the corrected assembly?

*RE: Figure S3 shows no local discrepancy was found. In addition, the “linkage” module that we wrote and used to do the verification is freely available and documented on the github repository, alongside the scaffolder and the correction module.*

- I believe the section entitled “Comparisons with previous Ectocarpus sp. assemblies and validation of the instaGRAAL assembly” is misguided, and I believe the entire analysis needs to be reconsidered. I have 3 primary suggestions:

ESR should be used as the “reference” assembly. This is because ESR has a much more complete set of gene annotations. Therefore, one could try to map these annotations onto ESG or ESI as a measurement of accuracy. I don’t believe it is valid to map the less complete annotations from ESD, as is done in the current manuscript.

ESD should not be included in this analysis. A sufficient comparison to ESD and ESI is already covered in “Correcting the chromosome-level instaGRAAL assembly of the Ectocarpus sp. Genome”. If you want to include a ESD/ESG comparison, include it in that section.

QUAST should not be used here. QUAST requires a reference genome, and if you take my advice from the earlier, the reference would be ESR.

*RE: As described above, we decided to maintain the use of the v1 assembly as a ‘reference’. High-quality genomes are differentiated from draft genomes by their completeness (low number of gaps), low number of errors, high percentage of sequence assembled into chromosomes. Obviously, a v2 is better, and indeed compared with v2, the v1 is more ‘draft’ but we believe the v1 is good enough to use QUAST, especially given how it is the v1 that was used to perform the assembly.*

But the whole point of this with each other in these assemblies, one does not know which assembly is correct, and therefore using ESR as a reference in QUAST is invalid. Rather, as was done in the previous sections, one should use the linkage map as an independent validation set. For example, one can align ESR, ESG and ESI to the linkage map and see which assembly shows the most concordance. You currently make use of the linkage map in this section, but just as before, there is no figure or table to show these results. As another potential independent validation, one could also align the Hi-C data to ESR/ESG and compare the contact frequency maps.

*RE: As we discussed above, figure S3 shows the concordance between linkage groups and scaffolds. If there were local discrepancies, it would show in the form of linkage marker positions progressing and looping back.*

- “Comparison between the Ectocarpus sp. instaGRAAL and pseudochromosomal assemblies” should be incorporated into the end of “Convergence of the Ectocarpus sp. assembly towards 27 major scaffolds”. I am not sure it warrants its own section.

*RE: Done.*

- QUAST should be used to assess the human genome scaffolding success. In this scenario, the human reference would be used as the “reference” and the instaGRAAL scaffolds would be used as the “query”. This would provide a nice quantitative assessment to supplement the qualitative assessment provided in the manuscript.

*RE: QUAST features were added in Table 2 (genome fraction, number of misassemblies) in addition to genomic features.*

- A reference to Table S4 should be placed after the following sentence: “ Sequencing reads were processed using the hybrid MaSuRCA assembler (v3.2.9) [31], yielding 7,743 contigs representing 496 Mb”.

*RE: Done*

Furthermore, 15X coverage of long reads is insufficient and may explain the poor assembly quality. In the discussion, the authors state “This is only a problem if one chooses not to reconstruct every input contig within a newly formed scaffold with our correction procedure, i.e. one is distrustful of the initial input contigs. This was not the case for Ectocarpus sp. but could be argued for D. herbacea, where the de novo contigs generated from an hybrid assembly are more error-prone”. But there is no evidence that MaSuRCA assemblies are more “error-prone” because they are hybrid assemblies, and I have never seen such claims in the literature. Rather, high-quality MaSuRCA assemblies are regularly published.

*RE: The referee is correct, and we have modified the discussion as he/she proposes, i.e., saying that 15x coverage may be insufficient and may explain the lower quality of the assembly.*

- The section entitled “Integrating information from the Hi-C analysis with other types of data” seems unnecessary and out of scope for this particular manuscript.

*RE: this was indeed more a discussion for specialists, and a quick review of recent literature, but we think it could stay there, unless the referee or editor really strongly object against it.*

- The section entitled “Validation with the genetic map” is unclear. Firstly, I believe the word “pseudochromosome” is incorrectly used in place of “linkage group”. The authors state “It then finds best-matching pairs of pseudochromosomes/scaffolds by counting how many of these regions overlap from one set to the other”, but what is meant by “overlap”. How do Hi-C links overlap with marker pairs?

*RE: We have changed “pseudochromosomes” to “linkage groups” as requested. By “overlap”, we mean contiguous stretches of DNA that both belong to a single scaffold and lie between a pair of markers. This is how we draw a correspondence in a first pass.*

Minor Points

- The manuscript states “... we ran SALSA2 [33] and 3D-DNA on the same Ectocarpus sp. and Desmarestiaherbacea reference genome and Hi-C reads”. Again this is a misuse of the word “reference”. I believe SALSA2 and 3D-DNA scaffolded the ESD genome, which is not a reference, along with the Desmarestia genome presented in this paper, which is also not a reference genome.

- The manuscript states “InstaGRAAL was also more efficient than the genetic map in orienting scaffolds (Table S2)”. But I believe “efficient” should be replaced with “accurate”.

*RE: Done*

- The discussion section “Reference genome assembly based correction” is misleading. I believe this refers to instaGRAAL’s “refinement” step and has nothing to do with a reference genome.

*RE: Correct, we have modified the title accordingly.*

- In general, the writing throughout the manuscript needs to be improved, specifically in terms of grammar and organization. Here are some examples:

“The Hi-C scaffolder GRAAL (Genome Re-Assembly Assessing Likelihood from 3D) is a probabilistic tool that uses a Markov Chain Monte Carlo (MCMC) approach was able to generate the first chromosome-level assembly of an incomplete eukaryotic genome [13] by permuting DNA segments according to their contact frequencies until the most likely scaffold was reached (see also [15]).” - This sentence starts off in the present tense and ends in the past tense. It also looks like it is missing a few words between “approach” and “was able”.

The word “exploit” is over-used.

“The remainder of the cycles are devoted to intra-chromosomal refinement”. - Here, “are” should be “is”.

*RE: We have made the changes suggested. The manuscript was reviewed by a couple of native English speakers, who are also co-authors. Some inconsistencies were corrected.*

“99.4% of the sequences in the 1,018 contigs was integrated into the 27 largest scaffolds after instaGRAAL processing.” - Here, “was” should be “were”.

*RE: Corrected.*
